# Identification and structural characterization of three psychoactive substances, phenylpiperazines (*p*BPP and 3,4-CFPP) and a cocaine analogue (troparil), in collected samples

**DOI:** 10.1007/s11419-021-00597-4

**Published:** 2021-09-14

**Authors:** Magdalena Popławska, Elżbieta Bednarek, Beata Naumczuk, Agata Błażewicz

**Affiliations:** National Medicines Institute, 30/34 Chełmska Street, 00-725 Warsaw, Poland

**Keywords:** Phenylpiperazines, Chlorofluorophenylpiperazine, Bromophenylpiperazine, Cocaine analogue, Troparil, New psychoactive substance

## Abstract

**Purpose:**

New psychoactive substances (NPSs) still appear on the market, mainly due to their legal status. This situation indicates and alarms that permanent recognition of the designer drug scene should be conducted. In this paper, we describe the detection of three psychoactive substances in samples collected from drug users.

**Methods:**

Qualitative characterization was performed using liquid chromatography–high-resolution tandem mass spectrometry with a quadrupole time-of-flight analyzer, gas chromatography with mass spectrometry and nuclear magnetic resonance spectroscopy.

**Results:**

In this study, we reported the detection and structural elucidation of three psychoactive substances: 1-(4-bromophenyl)piperazine (*p*BPP), 1-(3-chloro-4-fluorophenyl)piperazine (3,4-CFPP) and methyl 8-methyl-3-phenyl-8-azabicyclo[3.2.1]octane-4-carboxylate (troparil).

**Conclusions:**

To the best of our knowledge, this is the first report that presents an identification methodology for these substances found in illegal products. Comprehensive characterization of the NPSs presented in this paper facilitates their detection and identification by forensic and clinical laboratories.

**Supplementary Information:**

The online version contains supplementary material available at 10.1007/s11419-021-00597-4.

## Introduction

By the end of 2020, approximately 830 new psychoactive substances (NPSs) had been reported to the European Monitoring Centre for Drugs and Drug Addiction (EMCDDA) through the Early Warning System (EWS). As many as 46 NPSs were detected for the first time in Europe in 2020. Piperazine derivatives rank far behind synthetic cannabinoids and cathinones on the recreational drug list in this classification. According to the EMCDDA, there were only 18 piperazines monitored by the end of 2020 [1, 2], with one being the subject of EU risk assessment and international control (1-benzylpiperazine, BZP) [3]. These piperazines were reported to the EMCDDA, mainly in 2005 and 2006. Then, their popularity waned in favor of other groups of NPSs. Surprisingly, *p*FPP (1-(4-fluorophenyl)piperazine) was detected recently in combination with a synthetic cannabinoid in seized plant material [4].

The chemical structures of piperazine derivatives can be divided into two classes: benzyl-substituted piperazines with BZP as the main representative and phenylpiperazines, such as TFMPP (1-(*m*-trifluoromethylphenyl)piperazine), MeOPP (1-(4-methoxyphenyl)piperazine), *p*FPP, *m*CPP (1-(3-chlorophenyl)piperazine) or *p*CPP (1-(4-chlorophenyl)piperazine).

Cocaine is the second most commonly consumed illicit drug in the EU, following cannabis [3]; however, the recreational use of cocaine derivatives has been proven to be extremely rare. In 2008, the first detection of 4-fluorotropacocaine (*p*FBT, 8-methyl-8-azabicyclo[3.2.1]oct-3-yl 4-fluorobenzoate) was reported to the EWS-EMCDDA. Two other analogues, RTI-111 (dichloropane, methyl 3-(3,4-dichlorophenyl)-8-methyl-8-azabicyclo[3.2.1]octane-2-carboxylate) and WIN 35428 (methyl 3-(4-fluorophenyl)-8-methyl-8-azabicyclo[3.2.1]octane-2-carboxylate), were formally notified by the EMCDDA in 2016 [5] and 2018 [6], respectively.

Here, we describe the identification of three newly distributed NPSs found among illegal products collected from drug users: 1-(4-bromophenyl)piperazine (*p*BPP) (compound **1**), 1-(3-chloro-4-fluorophenyl)piperazine (3,4-CFPP) (compound **2**), and methyl 8-methyl-3-phenyl-8-azabicyclo[3.2.1]octane-4-carboxylate (troparil) (compound **3**). Analytical techniques, such as gas chromatography–electron ionization-mass spectrometry (GC–EI-MS), liquid chromatography–electrospray ionization-quadrupole time-of-flight-tandem mass spectrometry (LC–ESI-QTOF-MS), and nuclear magnetic resonance (NMR) spectroscopy, were used for the unequivocal assignment of the new chemical structures appearing on the market.

## Materials and methods

### Materials and reagents

Samples were collected from drug users by the National Bureau for Drug Prevention in Warsaw. One product was presented as a white round tablet in a packaging labeled ‘Hyper’, and two products were presented as white powders in small plastic bags with labels ‘4-BP’ and ‘3,4-CFP’.

Methanol and acetonitrile (LC–MS grade) were purchased from Merck Millipore (LiChrosolv; Darmstadt, Germany); formic acid (LC–MS grade) was purchased from Honeywell (Metropolis, IL, USA); dimethylsulfoxide-*d*_6_ (DMSO-*d*_*6*_, 100% D) was purchased from Euriso-top (Gif-Sur-Yvette, France); methanol (CD_3_OD + 0.03% TMS, 99.80% D) was purchased from ARMAR ISOTOPES (Leipzig, Germany); and deuterium oxide (D_2_O, 99.9% D) was purchased from Cambridge Isotope Laboratories, Inc. (Andover, MA, USA). Doubly distilled water additionally purified in a Nanopure Diamond UV Deionization System from Barnstead (Dubuque, IA, USA) was used throughout.

### GC–EI-MS

GC–MS analysis was performed using a gas chromatograph coupled to a GCMS-TQ8040 mass spectrometer (Shimadzu, Kyoto, Japan) with a Zebron ZB-SemiVolatiles column (30 m × 0.25 mm, with a film thickness of 0.25 µm; Phenomenex, Torrance, CA, USA). The samples were injected in splitless mode. After injection, the split flow was stopped for 1 min and then raised to 50.7 mL/min. Helium was used as the carrier gas with a column flow rate of 1.0 mL/min and nitrogen as a collision gas. The initial temperature was set to 75 °C, held for 1 min, ramped up to 180 °C at a rate of 20 °C/min, held for 3 min, increased to 320 °C at 20 °C/min and held for 7 min (total time 26 min). Electron ionization (EI) was used with an ionization voltage of 70 eV and an ion source temperature of 230 °C. The injector was maintained at a temperature of 250 °C, and the GC–MS transfer line was maintained at 280 °C. The scan range was *m/z* 29–600, and the injection volume was 1 µL.

### LC–ESI-QTOF-MS/MS

Analyses were performed using an Ultimate 3000 high-performance liquid chromatograph system from Dionex (Thermo Fisher Scientific, Waltham, MA, USA) coupled to a micrOTOF-QII high-resolution tandem mass spectrometer with time-of-flight analyzer from Bruker Daltonik (Bremen, Germany). Chromatographic separations were carried out at 25 °C on a Hypersil GOLD C18 analytical column (100 × 2.1 mm, 3 μm particle size; Thermo Fisher Scientific) with a Hypersil GOLD guard column (10 × 2.1 mm, 3 µm particle size; Thermo Fisher Scientific). Linear gradient elution was applied with solvent A consisting of water/acetonitrile/formic acid (90/10/0.1, v/v/v) and solvent B consisting of methanol/acetonitrile/formic acid (90/10/0.1, v/v/v), and the flow rate set at 0.15 mL/min. The following programme was used: 0–2 min, 10% B; 2–7 min, 10–90% B; 7–10 min, 90% B; 10–12 min, 90–10% B; 12–14 min, 10% B. The diode array detector (DAD) was set with wavelengths ranging from 190 to 320 nm. The mass spectrometer provided a resolving power that can exceed 17 500 FWHM (full width at half maximum). MS conditions were as follows: ESI positive ion mode; capillary voltage, 4500 V; end plate offset, − 500 V; dry gas flow rate, 8.0 L/min; dry heater, 180 °C; MS data full scan mode (from *m/z* 50 to 1500). High mass accuracies were ensured by calibration of the TOF analyzer with a solution of sodium formate prior to each sample. An auto-MS/MS acquisition mode was used in fragmentation experiments. Collision-induced dissociation (CID) was performed with collision energy (CE) linearly ramped as a function of the *m/z* ratio. The CE gradient was as follows: for values from *m/z* 200 to 400, the CE increased from 20 to 25 eV for compounds **1** and **2** or from 35 to 40 eV for compound **3**. All data were processed by Compass 1.3 (Bruker Daltonik).

### NMR spectroscopy

The NMR spectra were recorded at 298 K on a Varian VNMRS-500 spectrometer (Varian, Inc., Palo Alto, CA, USA) operated at 499.8 and 125.7 MHz for ^1^H and ^13^C, respectively. The spectrometer was equipped with an inverse ^1^H{^31^P–^15^N} 5 mm Z-SPEC Nalorac IDG 500-5HT probe with an actively shielded *z*-gradient coil. The high-power ^1^H and ^13^C *π*/2 pulses were 7.6 and 11.6 μs, respectively. The NMR experiments (^1^H and ^13^C NMR spectra, ^1^H–^1^H COSY, ^1^H–^13^C HSQC and ^1^H–^13^C HMBC) were run using the standard Varian pulse sequences (for detailed parameters see Electronic Supplementary Material 1—ESM_1).

### Sample preparation

Samples were micronized (in the case of tablets) and homogenized before testing. For GC–EI-MS, approximately 1 mg of each powder was dissolved in acetonitrile. For LC–QTOF-MS/MS analysis, samples were dissolved in a 1:1:1 (*v/v/v*) mixture of water/methanol/acetonitrile. In both cases, samples were dissolved with the assistance of ultrasonication for 10 min and then filtered by Whatman 0.2 μm pore size polytetrafluoroethylene (PTFE) filter media (GE Healthcare, Chicago, IL, USA). If necessary, the filtrates were further diluted to a suitable concentration.

For NMR analysis, several milligrams of powder were dissolved in 0.7 mL of DMSO-*d*_*6*_ or CD_3_OD. The solutions were transferred to 5 mm NMR tube.

## Results

Targeted compounds could not be easily identified by matching respective MS/MS spectra with reference standards or with those described in the databases and in scientific papers because, although they were not new compounds, they have not been known and used as psychoactive substances before. Hence, no analytical data were found during our identification. The unknown compounds were analyzed, and their structures were elucidated using complementary GC–EI-MS, LC–ESI-QTOF-MS and NMR methods.

Accurately measured masses, exact (theoretical) masses, errors, relative intensities and elemental compositions for precursor ions and product ions are displayed in Table 1.

**Table 1:** Cumulative data of product ion spectra obtained from precursor ions of identified substances

Compound name	Precursor ion[M + H] ^+^ Productions (*m/z*)	Intensity [%]	Predicted formula	Theoretical values (*m/z*)	Error [mDa]	Error [ppm]	Electron configuration
Compound **1** (*p*BPP)	**241.0329**	93.5	**C**_**10**_**H**_**14**_**BrN**_**2**_	241.0335	0.6	2.4	Even
	224.0078	1.1	C_10_H_11_BrN	224.0069	− 0.8	− 3.8	Even
	211.9942	0.4	C_8_H_9_BrN_2_	211.9944	0.1	0.7	Odd
	197.9908	35.2	C_8_H_9_BrN	197.9913	0.5	2.4	Even
	162.1148	40.8	C_10_H_14_N_2_	162.1151	0.4	2.4	Odd
	145.0892	2.9	C_10_H_11_N	145.0886	− 0.6	− 3.9	Odd
	132.0814	5.2	C_9_H_10_N	132.0808	− 0.6	− 4.7	Even
	120.0813	74.3	C_8_H_10_N	120.0808	− 0.5	− 4.5	Even
	119.0731	32.2	C_8_H_9_N	119.0730	− 0.1	− 0.9	Odd
Compound **2** (3,4-CFPP)	**215.0745**	100.0	**C**_**10**_**H**_**13**_**ClFN**_**2**_	215.0746	0.1	0.5	Even
	198.0472	3.0	C_10_H_10_ClFN	198.0480	0.9	4.4	Even
	180.1055	1.2	C_10_H_13_FN_2_	180.1057	0.3	1.5	Odd
	172.0326	65.1	C_8_H_8_ClFN	172.0324	− 0.2	− 1.1	Even
	163.0785	1.4	C_10_H_10_FN	163.0792	0.6	4.0	Odd
	137.0639	25.6	C_8_H_8_FN	137.0635	− 0.3	− 2.5	Odd
Compound **3** (Troparil)	**260.1644**	26.4	**C**_**16**_**H**_**22**_**NO**_**2**_	260.1645	0.1	0.3	Even
	228.1383	27.2	C_15_H_18_NO	228.1383	0.0	0.1	Even
	210.1279	13.9	C_15_H_16_N	210.1277	− 0.2	− 0.8	Even
	200.1425	9.4	C_14_H_18_N	200.1434	0.9	4.3	Even
	197.0947	7.6	C_14_H_13_O	197.0961	1.4	7.0	Even
	186.1269	13.0	C_13_H_16_N	186.1277	0.8	4.3	Even
	184.1119	49.4	C_13_H_14_N	184.1121	0.2	0.8	Even
	143.0855	63.2	C_11_H_11_	143.0855	0.0	0.1	Even
	141.0692	11.1	C_11_H_9_	141.0699	0.7	4.9	Even
	131.0501	93.6	C_9_H_7_O	131.0491	− 0.9	− 7.2	Even
	129.0705	100.0	C_10_H_9_	129.0699	− 0.6	− 4.9	Even
	128.0620	36.3	C_10_H_8_	128.0621	0.1	0.5	Odd
	117.0689	13.0	C_9_H_9_	117.0699	1.0	8.1	Even
	115.0529	14.6	C_9_H_7_	115.0542	1.4	11.8	Even
	105.0708	14.3	C_8_H_9_	105.0699	− 0.9	− 8.5	Even
	103.0545	33.3	C_8_H_7_	103.0542	− 0.3	− 2.6	Even
	96.0808	45.3	C_6_H_10_N	96.0808	− 0.0	− 0.2	Even
	91.0543	51.7	C_7_H_7_	91.0542	− 0.1	− 1.1	Even
	84.0807	41.3	C_5_H_10_N	84.0808	0.1	0.9	Even
	82.0655	24.9	C_5_H_8_N	82.0651	− 0.4	− 4.5	Even

Bold values indicate precursor ions

In the GC–EI-mass spectra of these compounds, signals of molecular ions were intense enough to be detected, but the spectra did not correspond with any available analytical data or databases. LC–ESI-QTOF-MS/MS provides very accurate mass information, isotopic patterns and MS/MS fragmentation patterns that allow for unambiguous assessment of empirical formulas or even chemical structures. Therefore, this technique is extremely useful in the identification of unknown substances. The mass accuracy for MS and MS/MS scans for compounds described in this paper and their product ions was high (within 5 ppm), although there were some exceptions for which the error was larger. The electron configuration was always even (EE) for precursor ions but even or odd (OE) for product ions. The structural identifications of fragment ions were based on theoretical calculations and comparisons with assignments of analogous fragments arising from fragmentations of other compounds. This study did not investigate fragmentation mechanisms. In some cases, ion formation required hydrogen rearrangements, but for the simplicity of the description, this was not specified each time it occurred.

Nevertheless, one of the problems that could not be resolved by mass spectrometric experiments was the assignment of substituents to *ortho, meta* or *para* positions on a phenyl ring. This information could be obtained by NMR experiments. The structures of the investigated compounds were determined by the interpretation of 1D and 2D NMR spectra: ^1^H, ^13^C, COSY, HSQC and HMBC. The presence of oxygen, nitrogen, chlorine and bromine atoms in the investigated compounds was confirmed by their influence on the NMR proton and carbon resonances and by the MS method.

### Identification of compound 1: *p*BPP

#### GC–EI-MS

**Fig. 1:**
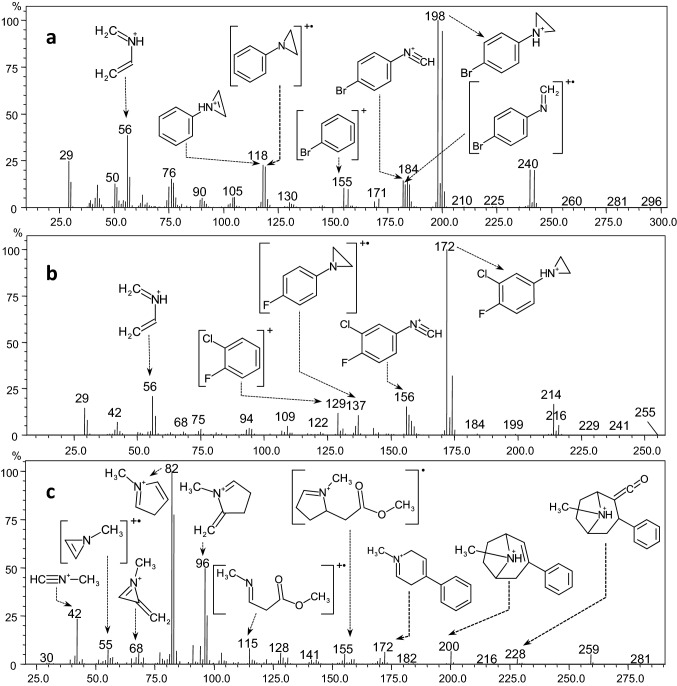
Gas chromatography–electron ionization mass spectra of *p*BPP (compound **1**) (**a),** 3,4-CFPP (compound **2**) (**b**), and troparil (compound **3**) (**c**) with proposed ion annotations

On the GC–MS chromatogram, a peak for compound **1** was observed at 11.9 min (Fig. S19a in ESM_2). The molecular ion at *m/z* 240 had a bromine isotope pattern (Fig. 1a). A base peak (M-42)^+^ (*m/z* 198) was formed by cleavage within the piperazine ring and the loss of the C_2_H_4_N fragment. Another characteristic ion indicating the presence of a piperazine ring (C_3_H_6_N^+^) was observed at *m/z* 56. Ions containing a phenyl ring substituted with a bromine atom appeared as doublets and were observed at *m/z* 155/157 (C_6_H_4_Br^+^), *m/z* 182/184 (C_7_H_5_NBr^+^) and *m/z* 183/185 (C_7_H_6_NBr^**·**+^). An isotope pattern for the product ion at *m/z* 119 and a characteristic mass loss of 79 Da from the base peak confirmed the release of bromine.

#### LC–ESI-QTOF-MS/MS

**Fig. 2:**
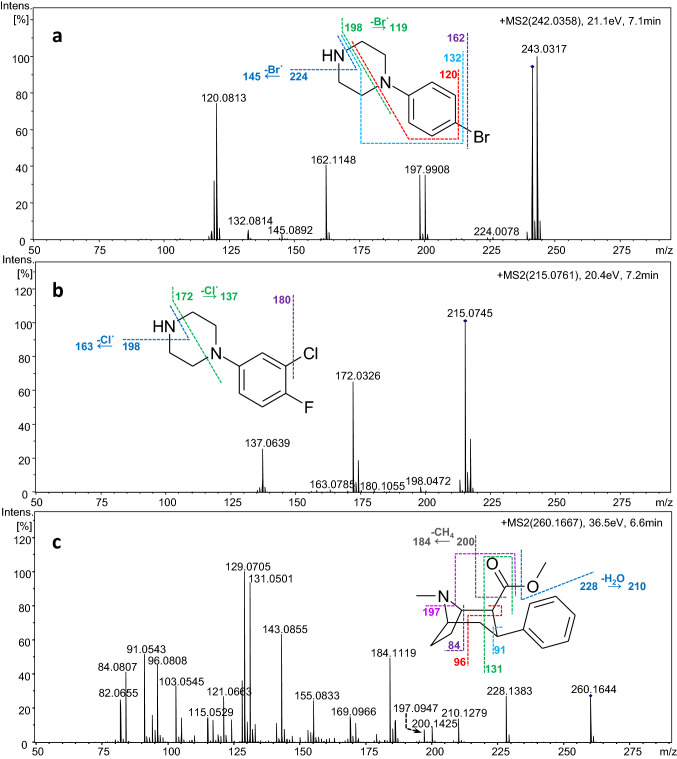
Product ion spectra of *p*BPP (compound **1**) (**a**), 3,4-CFPP (compound **2**) (**b**), and troparil (compound **3**) (**c**) with assigned fragmentation patterns

The LC–ESI-QTOF-MS/MS spectrum of compound **1** at a retention time of 7.1 min, displayed two isotopic ion signals of similar intensity [M + H]^+^ and [M + 2 + H]^+^ at *m/z* 241.0329 and *m/z* 243.0317, respectively, which indicated the presence of a bromine substituent (Fig. 2a). The monoisotopic ion at *m/z* 241.0329 corresponded to the protonated molecule C_10_H_14_BrN_2_^+^ (calculated: 241.0335, error 2.4 ppm). CID experiments with this precursor ion resulted in two main fragmentation pathways. The ions at *m/z* 224.0078 and 197.9908, with an isotopic pattern suggesting the presence of bromine in their structures, were produced via the loss of ammonia from the piperazine ring (∆ 17.0251, NH_3_) followed by the loss of C_2_H_2_. Homolytic cleavage of a bromine group, which yielded the OE ion (C_10_H_14_N_2_^**·**+^) at *m/z* 162.1148, initiated the second dissociation pathway. It included product ions at *m/z* 145.0892 corresponding to the loss of NH_3_ and at *m/z* 119.0731 derived from further cleavage of the C_2_H_2_ moiety (∆ 26.0161 Da). However, the most intense product ion in the MS/MS spectrum at *m/z* 120.0813 (C_8_H_10_N^+^) was formed by the loss of the C_2_H_4_N fragment from the piperazine ring and a bromine moiety from the phenyl ring.

#### NMR spectroscopy

The ^1^H NMR spectrum of compound **1** consisted of four signals (12 H); signals at 3.39 and 3.36 ppm represented eight aliphatic protons belonging to the piperazine ring and four aromatic protons gave signals at 7.39 and 6.95 ppm (Table 2). In the ^13^C NMR spectrum of compound **1**, four signals attributed to four pairs of equivalent carbon atoms were present: two signals from the CH_2_ groups of the piperazine ring and two from the CH groups of aromatic carbon atoms. The other two signals belonged to quaternary carbon atoms of the aromatic ring (Table 2). The presence of the AA′BB′ spin–spin coupling pattern in the aromatic part of the ^1^H NMR spectrum and the presence of four signals in the aromatic range of the ^13^C NMR spectrum (two signals of the CH and two signals of the quaternary carbon atoms) confirmed the *para* substitution of the phenyl ring. Bromine substitution was confirmed by the MS method. NMR spectra of compound **1** are presented in Figs S1–S6 in ESM_1.

**Table 2:** Experimental 1 dimensional (D) and 2D nuclear magnetic resonance (NMR) spectroscopic data of compound **1** in CD_3_OD, 25 ºC

Numbering of atom position see figure	^1^H NMR *δ* [ppm] (multiplicity, number of protons *J*_HH_ [Hz])	^13^C NMR *δ* [ppm]	^1^H–^13^C HMBC	
**1** (C)	–	150.8	–	
**2/6** (CH)	6.95 (*m*, 2H)	119.8	C2/6, C4	
**3/5** (CH)	7.39 (*m*, 2H)	133.1	C1, C3/5, C4	
**4** (C)	–	114.4	–	
**8/12** (CH_2_)	3.39 (*m*, 4H)	47.6	C1, C8/12, C9/11	
**9/11** (CH_2_)	3.36 (*m*, 4H)	44.7	C8/12, C9/11	

δ chemical shift [ppm], ^*1*^*H–*^*13*^*C HMBC* heteronuclear multiple bond correlation, *m* multiplet

Finally, compound **1** was determined to be 1-(4-bromophenyl)piperazine (*p*BPP).

### Identification of compound 2: 3,4-CFPP

#### GC–EI-MS

Compound **2** appeared at 10.8 min on the GC–MS chromatogram (Fig. S19b in ESM_2), with the molecular ion at *m/z* 214 displaying a chlorine isotope pattern (Fig. 1b). A base peak at *m/z* 172 (C_8_H_8_ClFN) indicated C_2_H_4_N loss. Similar to compound **1**, the signal of the C_3_H_6_N^+^ ion at *m/z* 56 suggested the presence of a piperazine moiety. The elimination of a chlorine moiety from the base peak generated the signal at *m/z* 137, while the neutral loss of CH_4_ yielded the ion at *m/z* 156 (C_7_H_4_ClFN). A chlorofluorophenyl cation was most likely observed at *m/z* 129 (C_6_H_3_FCl^+^).

#### LC–ESI-QTOF-MS/MS

Compound** 2** eluted at a retention time of 7.2 min with the LC method described above. On the mass spectrum of the chromatographic peak, a signal derived from protonated molecular ion [M+H]^+^ at *m/z* 215.0745 was observed with an isotopic pattern indicating the presence of one chlorine atom in the molecule. The predicted chemical formula was C_10_H_13_ClFN_2_^+^ (calculated *m/z* 215.0746, error 0.5 ppm). The MS/MS spectrum of compound **2** is presented in Fig. 2b. As with *p*BPP, two fragmentation pathways involving EE and OE ion series were observed. The first product ion at *m/z* 198.0472 was generated by the loss of ammonia from the piperazine ring. Subsequent cleavage of C_2_H_2_ from the remaining nitrogen formed the most intense product ion at *m/z* 172.0326, as seen in the EI-MS spectrum. The second CID pathway included odd electron product ions. It began with chlorine radical loss (Cl^**·**^) to yield a stable aromatic OE ion at *m/z* 180.1055. The next fragmentation steps were similar to those of the first CID pathway: they involved loss of ammonia followed by the loss of C_2_H_2_, which gave OE ions at *m/z* 163.0785 and 137.0639, respectively.

#### NMR spectroscopy

To determine the positions of halogen substituents (Cl and F) on the phenyl ring, NMR experiments were performed. The ^1^H NMR spectrum of compound **2** consisted of four signals (11 H) representing eight aliphatic protons on the piperazine ring and three aromatic protons (Table 3). In the ^13^C NMR spectrum of compound **2**, eight signals were attributed to: two pairs of equivalent carbon atoms from the CH_2_ groups of the piperazine ring, three carbon atoms of the CH groups and three quaternary carbon atoms of the aromatic ring (Table 3). Five signals for aromatic carbon atoms in the ^13^C NMR spectrum and three aromatic proton signals in ^1^H NMR spectra were coupled with the fluorine atom, and the coupling constants are presented in Table 3. The ABX spin–spin coupling pattern in the ^1^H NMR spectrum and the presence of six signals in the aromatic range of the ^13^C NMR spectrum (three signals for CH and three signals for quaternary carbon atoms) meant that the phenyl ring in the structure of compound **2** was doubly substituted. The signal at 149.0 ppm was attributed to the carbon atom at position C1 based on the presence of a cross-peak at 3.37 ppm/149.0 ppm in the ^1^H{^13^C}HMBC spectrum. Moreover, the coupling constant for carbon atom C1 with fluorine was equal to *J*_CF_ = 2.9 Hz, which suggested that the fluorine substituent is in the *para*-position (C4 at 154.3 ppm). The next quaternary carbon atom at 122.0 ppm was also coupled with a fluorine atom with a coupling constant equal to *J*_CF_ = 18.3 Hz, which is a typical value for carbon–fluorine coupling through two bonds. This suggested that the chlorine atom is substituted in the *meta* position (C3). The presence of chlorine and fluorine atoms was confirmed by the MS method. The NMR spectra of compound **2** are presented in Figs S7–S14 in ESM_1.

**Table 3:** Experimental 1D and 2D NMR spectroscopic data of compound **2** in CD_3_OD, 25 °C

Numbering of atom position see figure	^1^H NMR *δ* [ppm] (multiplicity, number of protons *J*_*HH*_, *J*_*HF*_ [Hz])	^13^C NMR *δ* [ppm](*J*_CF_ [Hz])	^1^H–^13^C HMBC	
**1** (C)	–	149.0(*J*_CF_ = 2.9 Hz)	–	
**2** (CH)	7.13 (*dd*, 1H, *J*_HH_ = 3.6 Hz, *J*_HF_ = 6.1 Hz)	120.1	C4, C6	
**3** (C)	–	122.0 (*J*_CF_ = 18.3 Hz)	–	
**4** (C)	–	154.3 (*J*_CF_ = 242.0 Hz)	–	
**5** (CH)	7.14 (*dd*, 1H, *J*_HH_ = 9.0 Hz, *J*_HF_ = 9.0 Hz)	117.9 (*J*_CF_ = 21.8 Hz)	C1, C3, C4	
**6** (CH)	6.90 (*ddd*, 1H, *J*_HH_ = 9.0, 3.8 Hz, *J*_HF_ = 2.8 Hz)	118.3 (*J*_CF_ = 6.9 Hz)	C4, C2	
**8/12** (CH_2_)	3.37 (*s*, 8H)	48.1	C1, C8/12, C9/11	
**9/11** (CH_2_)		44.7		

δ chemical shift [ppm], *dd* doublet of doublets*, ddd* doublet of doublets of doublets, *s* singlet ^*1*^*H–*^*13*^*C HMBC* heteronuclear multiple bond correlation, *J*_*HH*_ proton–proton coupling constant [Hz], *J*_*HF*_ proton–fluorine coupling constant [Hz], *J*_*CF*_ carbon–fluorine coupling constant [Hz]

Finally, compound **2** was determined to be 1-(3-chloro-4-fluorophenyl)piperazine (3,4-CFPP).

### Identification of compound 3: troparil

#### GC–EI-MS

Compound** 3** appeared at 12.5 min on the GC–MS chromatogram (Fig. S19c in ESM_2) with a molecular ion at *m/z* 259 (Fig. 1c). The EI-mass spectrum of compound **3** and a brief description has already been published [7]; however, we add its GC–MS spectrum with proposed ion annotations in this paper to collect and present complete analytical data of all characterized compounds.

#### LC–ESI-QTOF-MS

The peak of compound **3** was detected at a retention time of 6.6 min under the experimental chromatographic conditions. The protonated molecular ion at *m/z* 260.1644 was displayed in the mass spectrum, corresponding to the suggested formula C_16_H_22_NO_2_^+^ (calculated: 260.1645, error 0.3 ppm). A collision energy gradient had to be optimized to obtain more product ions and of higher intensity (Fig. S20 in ESM_2). The product ion in the MS/MS spectrum of compound **3** at *m/z* 228.1383 (C_15_H_18_NO^+^) was most likely due to an acylium ion generated by ester bond cleavage with loss of the CH_3_OH moiety. Elimination of the carbonyl group produced the product ion at *m/z* 200.1425 corresponding to the phenyltropane moiety. The subsequent loss of CH_4_ yielded the ion at *m/z* 184.1119. Other fragmentation pathways that started from the ion at *m/z* 228.1383 involved dehydration, which resulted in the ion at *m/z* 210.1279, or a neutral loss of methylamine (31 Da), which formed the product ion at *m/z* 197.0947. Further cleavage of a tropane ring generated the most abundant ions in the MS/MS spectrum derived from the phenyl moiety substituted with unsaturated hydrocarbon chains, which were the residues of a tropane ring. A signal at *m/z* 143.0855 corresponded to the C_11_H_11_^+^ fragment. The loss of CH_2_ yielded the ion at *m/z* 129.0705 (C_10_H_9_^+^). Low-mass product ions appearing at *m/z* 96.0808, *m/z* 84.0807 and *m/z* 82.0655 were fragments containing pyrrole or partially saturated pyrrole substructures derived from the pyrrolidine ring of tropane, while the ion appearing at *m/z* 91.0543 was the tropylium cation C_7_H_7_^+^. Apart from the OE product ion at *m/z* 128.0620, which was formed by the elimination of CH_3_• radical, all of the fragments generated via CID experiments were even-electron ions.

Some steps of CID fragmentation of compound **3** were analogous to those of other compounds with tropane groups, such as cocaine [8].

To confirm the structure proposed based on LC–QTOF-MS/MS analysis, NMR experiments were conducted.

#### NMR spectroscopy

The NMR data for compound **3** are collected in Table 4. The ^1^H NMR spectrum of compound **3** consisted of signals belonging to 16 aliphatic and 5 aromatic protons (two singlets for CH_3_ groups, ten multiplets (10 H) forming a spin–spin coupling pattern in the range 1.9–4.3 ppm and signals for one aromatic system AA′BB′C (phenyl group)). Moreover, there was a broad singlet from one proton that could be assigned to an NH^+^ group based on its chemical shift (9.4 ppm).

**Table 4:** Experimental 1D and 2D NMR spectroscopic data of compound **3** (troparil HCl) in DMSO, 25ºC

Numbering of atom position (see figure)	^1^H NMR *δ* [ppm] (multiplicity, number of protons *J*_*HH*_ [Hz])	^13^C NMR *δ* [ppm]	^1^**H-**^**1**^H COSY	^1^**H-**^**13**^C HMBC	
**1** (CH)	4.10 (*d,* 1H, *J*_*HH*_ = 7.5 Hz)	63.2	H7β, H2	C10	
**2** (CH)	3.26 (*d*, 1H, *J*_*HH*_ = 9.0 Hz)	52.0	H3, H1	C10, C3	
**3** (CH)	3.34 (*overlapped,* 1H)	34.4	H4α, H4β, H2	C10, C14, C15/19, C1, C2, C4	
**4α** (½ CH_2_)	1.95 (*dd*, 1H, *J*_*HH*_ = 9.4, 14.5 Hz)	35.6	H3, H4β	-	
**4β** (½ CH_2_)	2.45 (*ddd*, 1H, *J*_*HH*_ = 7.4, 7.4, 14.8 Hz		H3, H5, H4α	C14, C5, C2, C3, C6	
**5** (CH)	3.87 *(dd*, 1H, *J*_*HH*_ = 7.2, 7.2 Hz)	61.4	H4β, H6β	C3	
**6α** (½ CH_2_)	1.85 (*ddd*, 1H, *J*_*HH*_ = 4.4, 10.7, 14.5 Hz)	26.0	H7α, H7β, H6β	-	
**6β** (½ CH_2_)	2.21 (*m*, 1H)		H5, H7α, H6α	C5, C4	
**7α** (½ CH_2_)	2.00 *(ddd*, 1H, *J*_*HH*_ = 4.7, 10.1, 13.6 Hz)	26.1	H6α, H6β, H7β	-	
**7β** (½ CH_2_)	2.31 (*m*, 1H)		H1, H6α, H7α	C1, C2	
**9** (N-CH_3_)	2.68 (*s*, 3H)	39.2	-	C1, C5	
**10** (C = O)	-	173.2	-	-	
**13** (O-CH_3_)	3.55 *(s*, 3H)	52.7	-	C10	
**14** (C)	-	142.0	-	-	
**15/19** (CH)	7.36 *(dd*, 2H, *J*_*HH*_ = 1.6, 7.4 Hz)	127.8	H3	C3, C15/19, C17	
**16/18** (CH)	7.32 *(dd*, 2H, *J*_*HH*_ = 7.1, 7.4 Hz)	129.0	-	C14, C16/18, C15/19	
**17** (CH)	7.23 *(td*, 1H, *J*_*HH*_ = 1.6, 7.1 Hz)	127.3	-	C15/19	
^+^NH	9.42 (*brs*, 1H)	-	H9 (N-CH_3_)	-	

*brs* broad singlet, ^*1*^*H-*^*1*^*H COSY* correlation spectroscopy, *δ* chemical shift [ppm], *d* doublet, *dd* doublet of doublets*, ddd* doublet of doublets of doublets, ^*1*^*H-*^*13*^*C HMBC* heteronuclear multiple bond correlation, *J*_*HH*_ proton-proton coupling constant [Hz], *m* multiplet*, s* singlet, *td* triplet of doublets

The ^13^C NMR spectrum consisted of fourteen signals, twelve of which, based on the ^1^H{^13^C}HSQC spectrum, were assigned to carbon atoms bearing protons: CH_3_ (two signals), CH_2_ (three signals) and CH (four signals for aliphatics and three in the aromatic region of the spectrum) groups. The other two signals belonged to quaternary carbon atoms. The NMR spectra of compound **3** are presented in Figs S15–S18 in ESM_1.

Finally, compound **3** was identified as 3-phenyl-8-methyl-8-azabicyclo[3.2.1]octane-2-carboxylic acid methyl ester.

## Discussion

Piperazine derivatives were originally synthesized and used as anthelmintic agents, primarily in veterinary settings. *N*-Phenylpiperazines have been investigated in a wide range of therapeutic applications. Due to the serotoninergic, dopaminergic and adrenergic activity of *N*-phenylpiperazines, the majority of these studies were focused on treatments of central nervous system disorders; however, patents are also describing the use of *N*-phenylpiperazine derivatives to treat, i.a., chronic painful conditions, chronic inflammatory states or obesity [9].

The misuse of certain piperazine derivatives (often known as ‘party pills’) started in New Zealand in the early 2000s [10, 11] and then became common in Europe. Combinations of piperazine analogues, such as BZP and TFMPP, were mainly ingested by drug users to mimic the psychoactive effects of MDMA (4-methylenedioxymethamphetamine, ‘Ecstasy’) [12]. Since many European countries introduced control over abused at that time piperazines and a boom in new legal stimulants—cathinones—occurred, the development of new piperazine derivatives was abandoned for a long time. Possibly due to the enactment of the generics law in Poland in 2018 and control of four NPS groups i.e., 2-phenylethylamines, cathinones, synthetic cannabinoids and fentanyl derivatives (benzodiazepines were added in 2019 and tryptamines in 2021), users again turned to the forgotten piperazines.

All compounds presented in this paper were identified for the first time and reported to the EWS-EMCDDA by NMI—troparil in 2018 [13] and *p*BPP and 3,4-CFPP in 2019 [14, 15].

*p*BPP (street names: 4-bromo-piperein, 4-BP, brein) has been found in powder-labeled ‘4-BP’. It is structurally related to *p*FPP and to *p*CPP, both formally reported to the EWS-EMCDDA in 2006. The only difference is the type of halogen substituent attached at the *para*-position of the phenyl ring. This is the first bromine-substituted phenylpiperazine identified as a recreational drug. In the case of NPSs from methcathinone group, which are also neurotransmitter releasers, structure–activity studies demonstrated that replacement of a fluorine or chlorine atom with a bromine atom resulted in a compound with more potency for release of both dopamine and serotonin. The lowest dopamine selectivity, which was an additional consequence of these halogen replacements, results in lower abuse potential in favor of empathogenic properties [16].

The second powder, marked as ‘3,4-CFP’, contained 3,4-CFPP (street names: kleferein, 3-chloro-4-fluoro-piperein, 3,4-CFP), which is the 4-fluorophenyl derivative of *m*CPP and the 3-chlorophenyl derivative of *p*FPP, which were both formally reported to the EWS-EMCDDA in 2005 and 2006, respectively. 3,4-CFPP is the second disubstituted phenylpiperazine detected in Europe after 1-(2,3-dichlorophenyl)-piperazine (2,3-DCPP), which was identified for the first time in 2015 in Spain [17]. The synthesis and anorexigenic properties of 3,4-CFPP have been described in a patent filed in 1972 [18].

Based on the chemical structures of the identified phenylpiperazines and their similarities to *m*CPP (the intake of which causes a significant increase in serotonin and a slight increase in dopamine [19]), *p*BPP and 3,4-CFPP are expected to be psychostimulants.

Since NMI notification of 3,4-CFPP through a National Focal Point, this compound was also identified in Slovenia in 2020 by the National Forensic Laboratory (NFL) [20]. Their GC–MS results were consistent with the data presented in this paper. The GC/MS spectrum of a kleferein isomer—2-Cl-3-FPP (recorded for reference material and shared by NFL)—indicated much weaker chlorine binding at the *ortho* position compared to the *meta* position, as the ion at *m/z* 179 (corresponding to chlorine elimination from a precursor ion) of moderate intensity in the spectrum of 2-Cl-3-FPP was not present in the spectrum of 3,4-CFPP [21].

Troparil (another name: WIN 35,065-2) was found in a sample of ‘Hyper’. Troparil is a synthetic derivative of cocaine and was synthesized during the 1970s to separate the stimulant and depressant actions of cocaine from its toxicity and dependence liability [22]. This compound was also used as a starting material or intermediate in the synthesis of several 3β-phenyltropane derivatives [7, 23, 24]. With a phenyl group directly attached to the tropane ring, troparil is a 3-phenyltropane. The absence of the carboxylate bridge between the two cyclic moieties, which is present in cocaine, was reported to be responsible for the lack of local anesthetic activity [25] and to produce strongly enhanced stimulant activity [22]. Troparil also shares structural similarities with compounds monitored by the EWS-EMCDDA: RTI-111 and WIN 35428.

As mentioned above, *p*BPP has not been previously described in the literature in the context of its use as a psychoactive compound, although its ^1^H and ^13^C NMR data in CDCl_3_ were published [26]. In the cited work, *p*BPP was synthesized and then used as a substrate for the synthesis of 1-(4-(4-bromophenyl)piperazin-1-yl)-2-((tert-butyldimethylsilyl)oxy)ethan-1-one. Therefore, it was decided to report, in this paper, the appearance of *p*BPP among the illegal samples and present NMR data obtained in CD_3_OD.

In addition, some ^1^H and ^13^C NMR data obtained for troparil in CDCl_3_ [27] and ^1^H NMR data obtained in benzene [28] are available in the literature. Nevertheless, the difficulties that may arise in preparing real samples for measurement, including the selection of an appropriate solvent, persuaded us to present complete NMR data for troparil hydrochloride in DMSO.

Access to NMR data for a given compound dissolved in different solvents facilitates its identification. Therefore, here, we present full NMR characterization of troparil and *p*BPP in common solvents used in pharmaceutical and forensic laboratories, data that have not been published in the literature. Thus, the presented data could help in the fast identification of these NPSs in future samples seized from illegal markets.

## Conclusions

In summary, two phenylpiperazines (*p*BPP and 3,4-CFPP) and one cocaine analogue (troparil) were identified in this study as novel psychoactive substances. These NPSs were detected in samples collected from users. Their structures were elucidated by LC–ESI-QTOF-MS/MS and GC–EI-MS as well as one- and two-dimensional (1D and 2D) NMR spectral analysis. The information obtained from 1D and 2D NMR experiments allowed determination of proton and carbon connection schemes in the studied compounds, and by considering molecular formulas obtained using LC–ESI-QTOF-MS/MS, their structures were determined. Some of the analytical data regarding troparil and *p*BPP have been published; however, this is the first comprehensive report that could be used for detection and identification of the characterized NPSs in drug samples by forensic and clinical laboratories.

Information on the identification of new compounds used as recreational drugs was reported by the NMI to the corresponding National Focal Point, which in turn sent an official notification to the EMCDDA; the drugs were then included in the European Drug Network Database, a European information system and database on new drugs.

## Supplementary Information

Below is the link to the electronic supplementary material.

Supplementary file1 (PDF 766 KB)

Supplementary file2 (PDF 182 KB)
