# Supplementary material for: Identification and structural characterization of three psychoactive substances, phenylpiperazines (pBPP and 3,4-CFPP) and a cocaine analogue (troparil), in collected samples
Source: Forensic Toxicol. 2021 Sep 14;40(1):132–43. doi: 10.1007/s11419-021-00597-4 (PMC9715470; doi:10.1007/s11419-021-00597-4)
Supplement: Supplementary file 1 — Supplementary file1 (PDF 766 KB) [file 11419_2021_597_MOESM1_ESM.pdf]

Electronic Supplementary Material **ESM_1**

Forensic toxicology

Identification and structural characterization of three psychoactive substances: phenylpiperazines (*p*BPP and 3,4-CFPP) and cocaine analogue (troparil), in collected samples

Magdalena Popławska[1*], Elżbieta Bednarek[1*], Beata Naumczuk[1], Agata Błażewicz[1]

[1]National Medicines Institute, 30/34 Chełmska Street, 00-725 Warsaw, Poland,

*Corresponding author e-mail address:

m.poplawska@nil.gov.pl (Magdalena Popławska)

e.bednarek@nil.gov.pl (Elżbieta Bednarek)

NMR spectroscopy

The NMR spectra were recorded at 298 K on Varian VNMRS-500 spectrometer (Varian, Inc., Palo Alto, CA, USA) operated at 499.8 and 125.7 MHz for ^{1}H and ^{13}C, respectively. The spectrometer was equipped with an inverse $^{1}H\{^{31}P\text{-}^{15}N\}$ the 5 mm Z-SPEC Nalorac IDG 500-5HT probe with an actively shielded z-gradient coil. The high power ^{1}H and ^{13}C $\pi/2$ pulses were 7.6 and 11.6 µs, respectively. The NMR experiments were run by using the standard Varian pulse sequence.

The ^{1}H NMR spectra and the ^{1}H dimension in 2D heteronuclear spectra were referenced internally to tetramethylsilane (TMS). The 1D $^{13}C\{^{1}H\}$ spectra and ^{13}C dimension in 2D heteronuclear spectra were referenced to the solvent (CD_3OD, δ_c = 49.0 ppm or DMSO, δ_c = 40.0 ppm), which were treated as a secondary standard.

A standard single-pulse experiment was used to acquire the ^{1}H NMR spectra using an 8000 Hz spectral window, 30° pulse width, an acquisition time of 4.0 s, relaxation delay of 1 s, and 64000 complex data points.

The 1D ^{13}C NMR spectra were run with a spectral range of 32000 Hz, 30° pulse width, an acquisition time of 1.0 s, a relaxation delay of 1 s, and 32000 complex data points.

^{1}H-^{1}H COSY spectra were run by using spectral widths 6000 Hz in both dimensions, 1024 complex points in t_2, 512 increments in t_{1}, 1 - 4 scans per increment, and a relaxation delay of 1 s.

For ^{1}H-^{13}C HSQC we used spectral widths 6000 Hz in F2 and 19000 Hz in F1, 1024 complex points in t_2, 512 complex points in t_{1}, 2 scans per increment, a relaxation delay of 1 s and ^{1}J(C,H) = 146 Hz.

^1H-^{13}C HMBC was performed using spectral widths 6000 Hz in F2 and 22500 Hz in F1, 1024 complex points in t_2, 512 complex points in t_1, 8 scans per increment, a relaxation delay of 1 s and $^nJ_{(C,H)}$ = 8 Hz.

The data was processed with linear prediction in t_1 followed by zero filling in both dimensions. Gaussian window functions were applied in both dimensions prior to Fourier transformation.

The signals in the ^1H and ^{13}C NMR spectra of studied compounds were assigned to the proton and carbon atoms in the appropriate structural fragments with the aid of the proton-proton coupling pattern and the proton-proton and proton-carbon correlations obtained from ^1H-^1H COSY, ^1H-^{13}C HSQC and ^1H-^{13}C HMBC experiments. The presence of oxygen, nitrogen, chlorine, bromine and fluorine ions in structures of studied compounds and the molecular formulas were determined on the basis of QTOF-MS results.

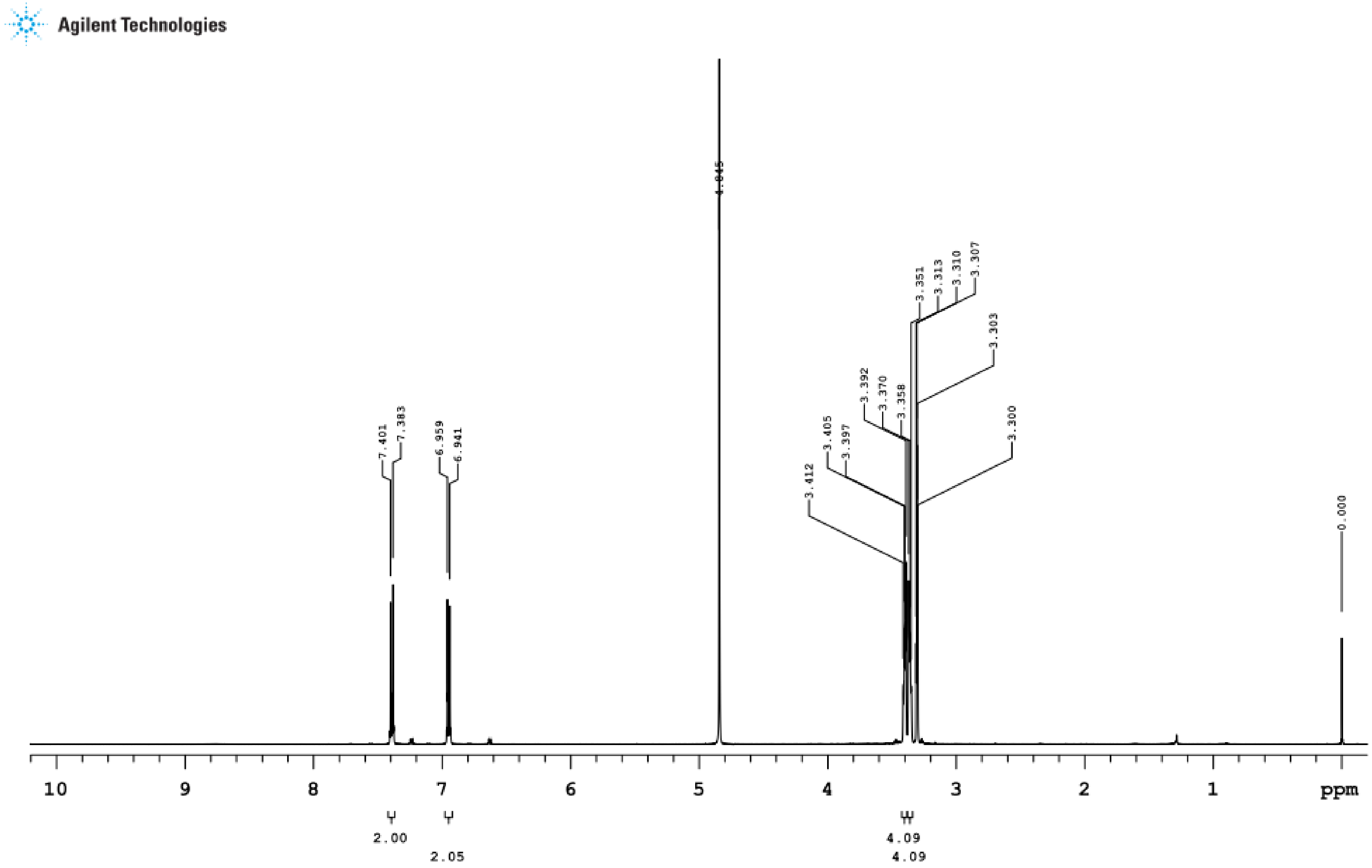

Fig. S1 The ^{1}H NMR spectrum of compound **1**

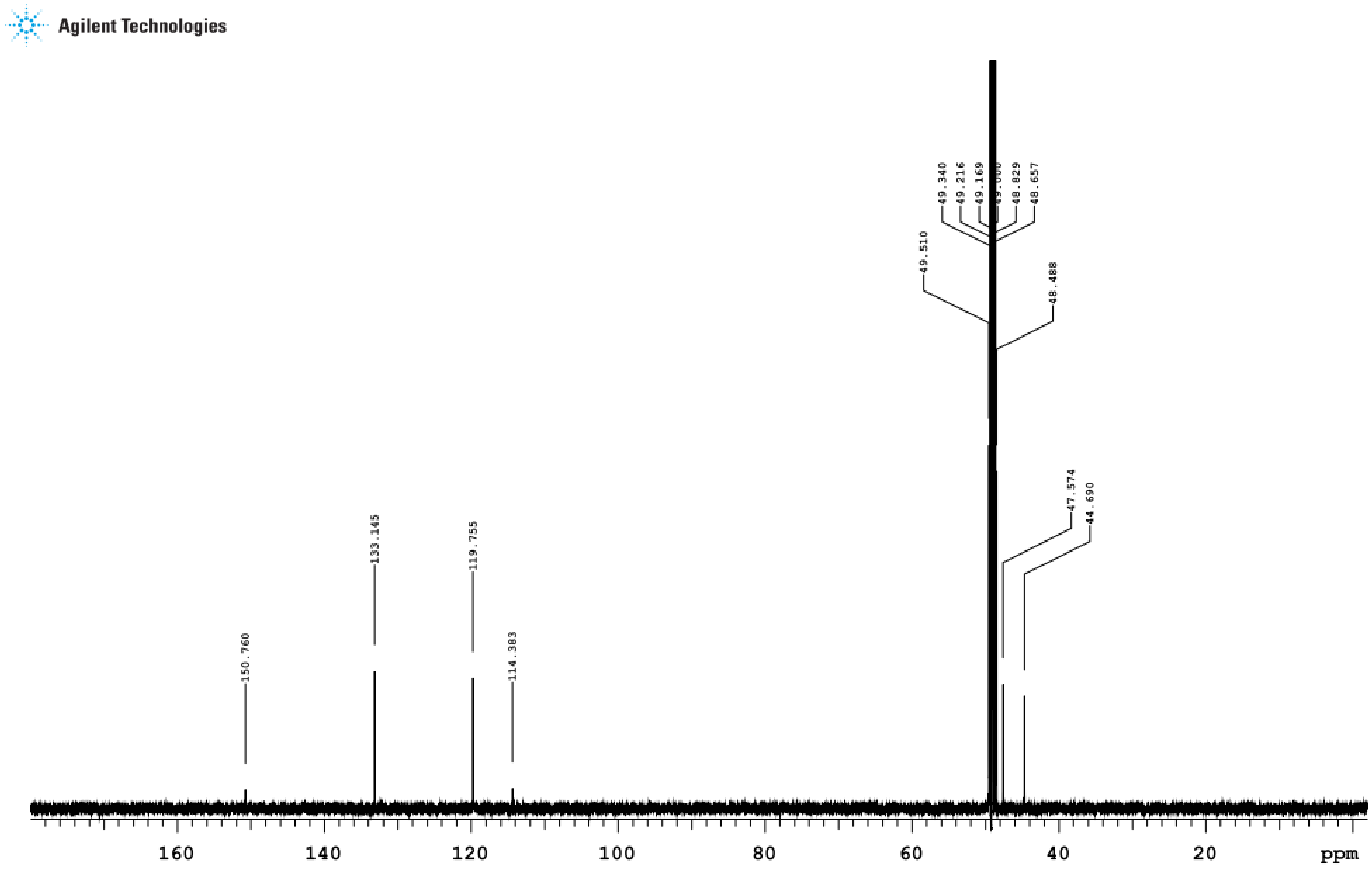

Fig. S2 The ^{13}C NMR spectrum of compound **1**

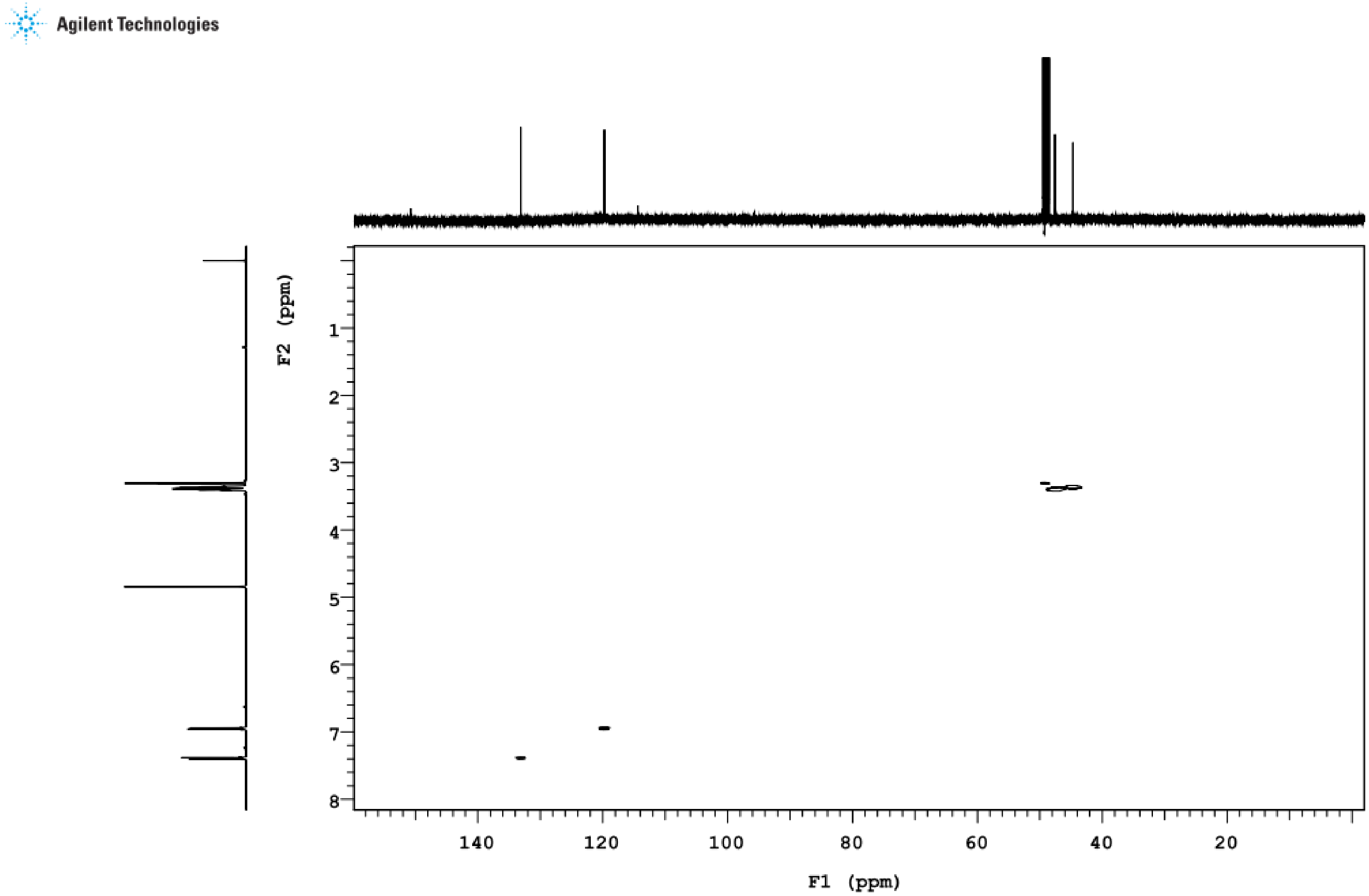

Fig. S3 The HSQC spectrum of compound **1**

Agilent Technologies

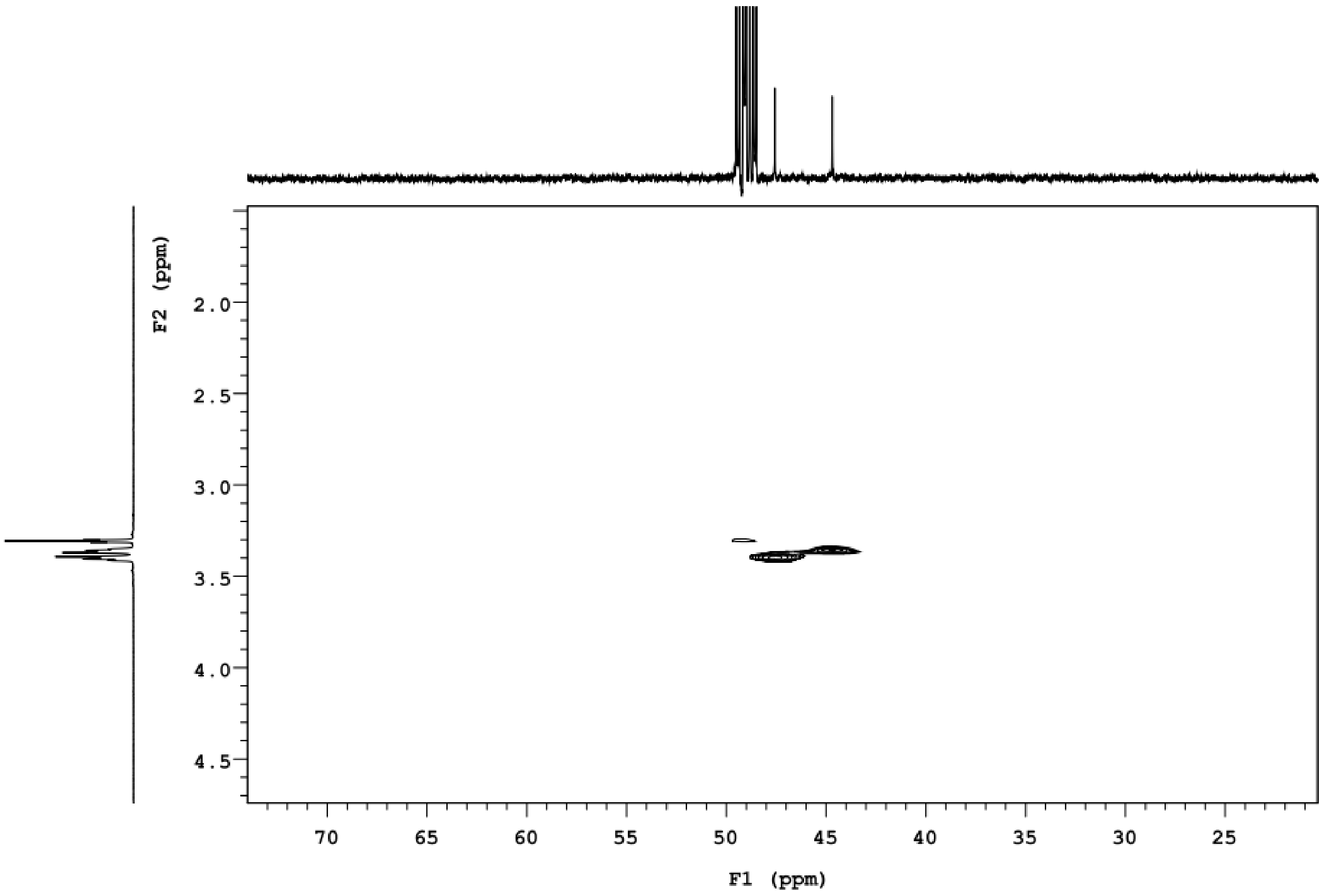

Fig. S4 The part of HSQC spectrum of compound **1**

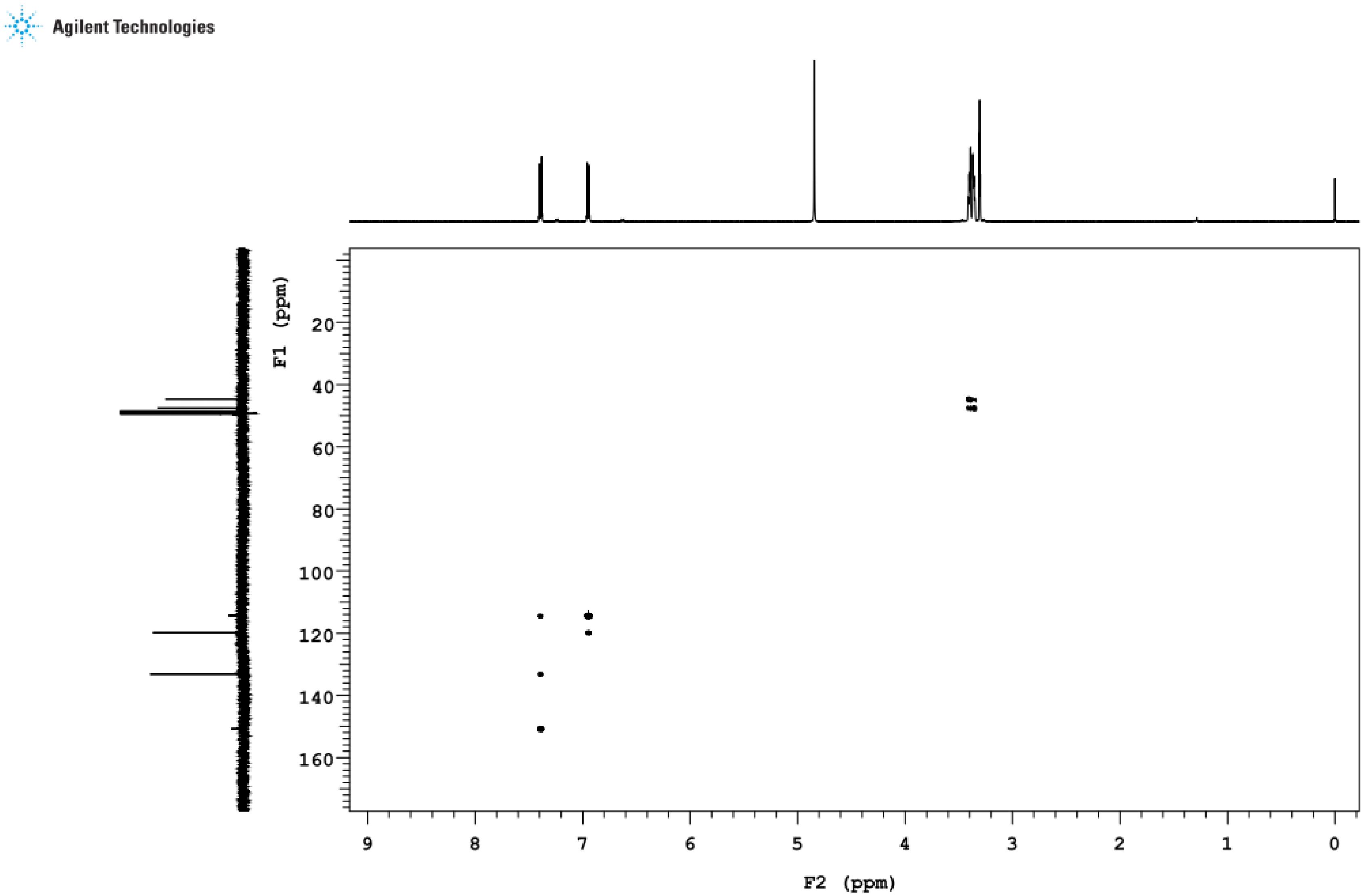

Fig. S5 The HMBC spectrum of compound **1**

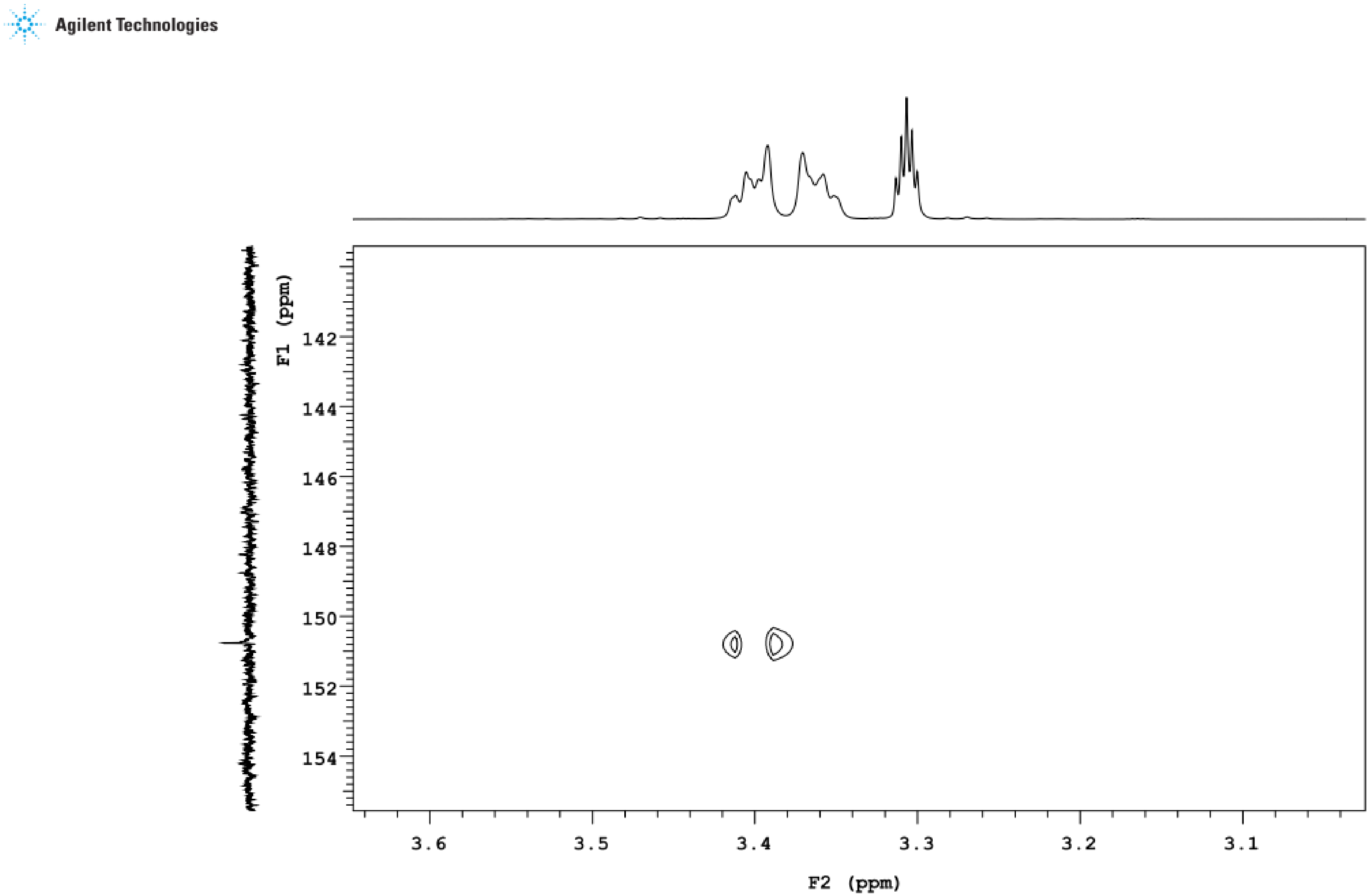

Fig. S6 The part of HMBC spectrum of compound **1**

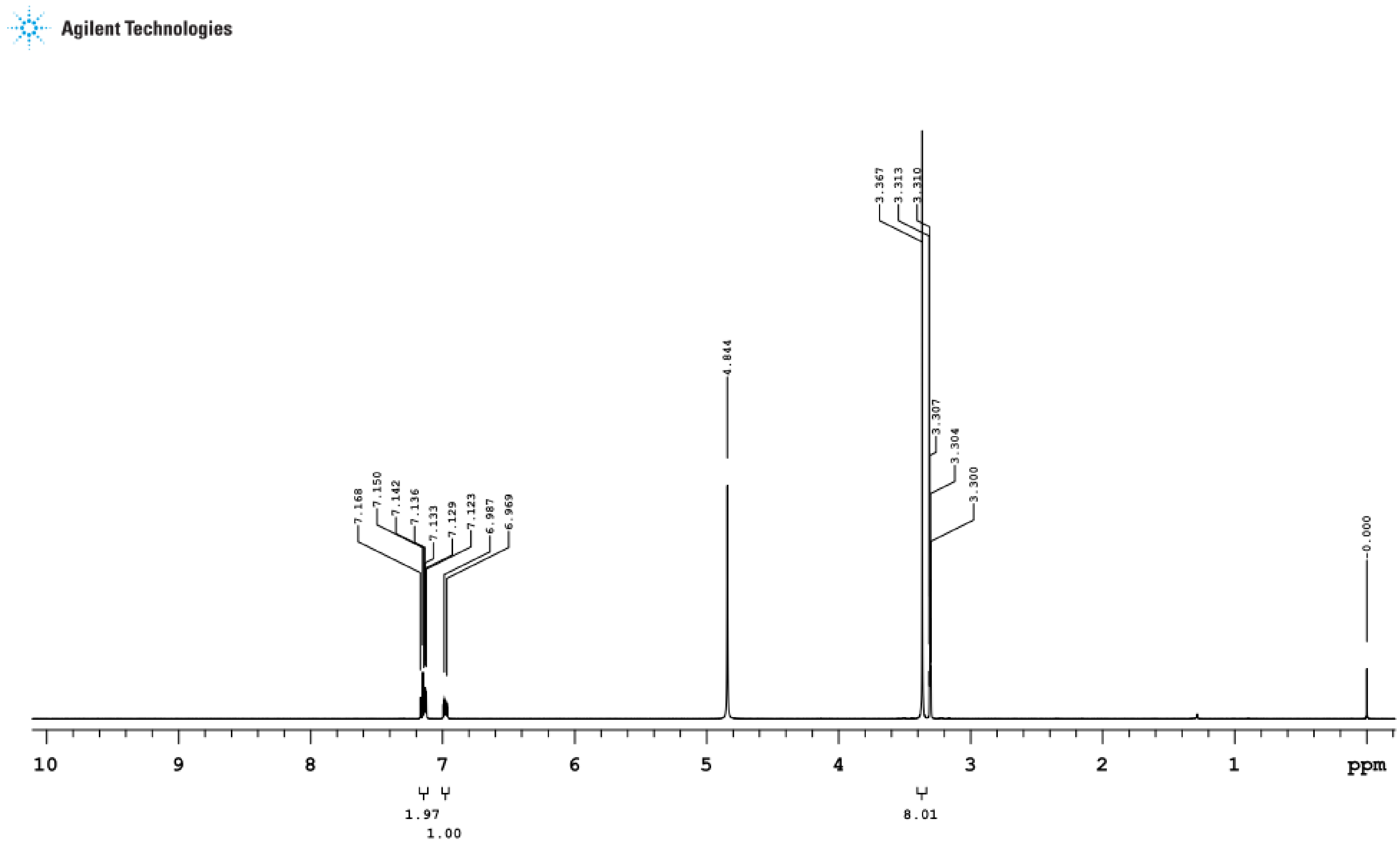

Fig. S7 The ^{1}H NMR spectrum of compound **2**

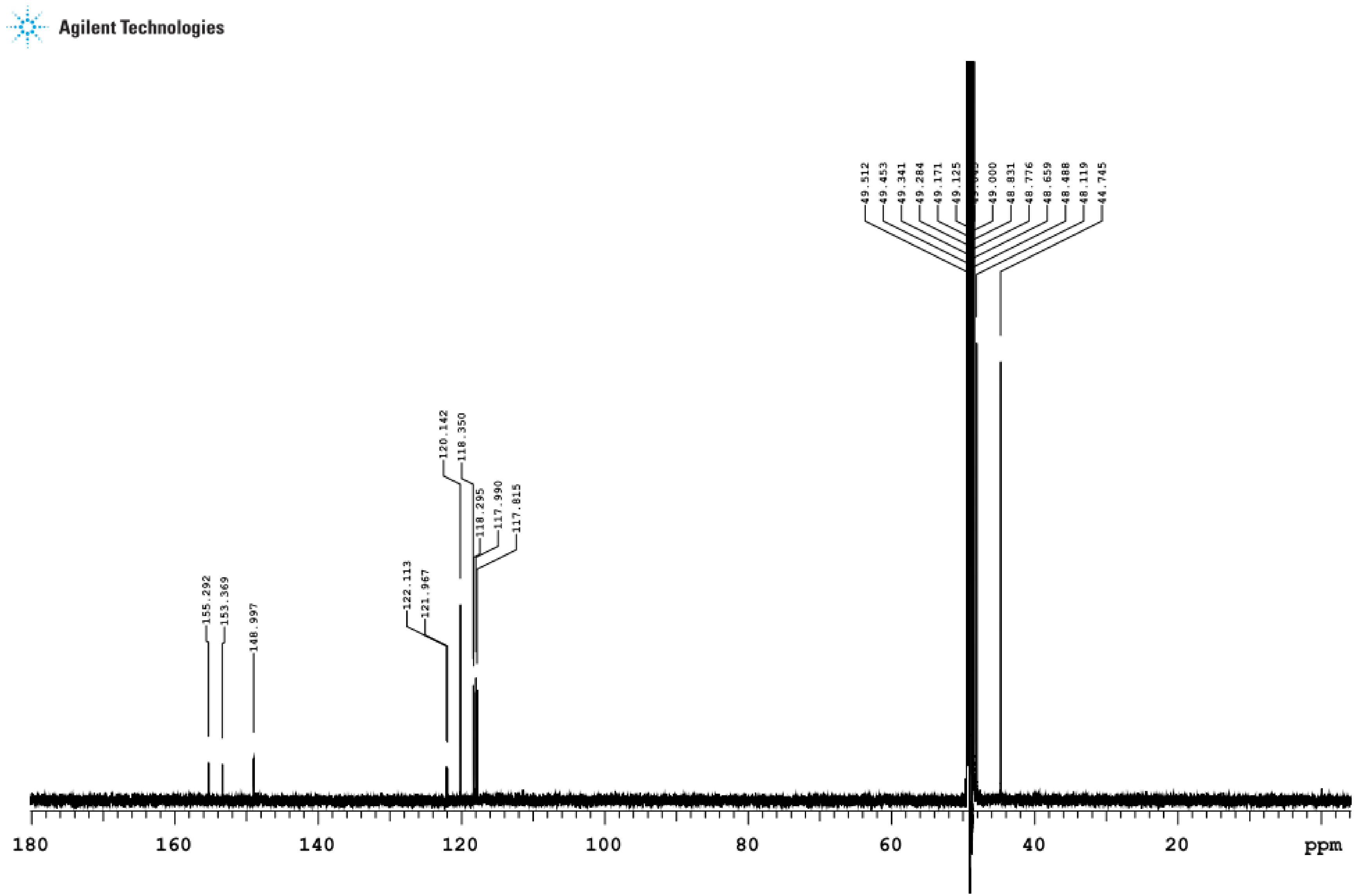

Fig. S8 The ^{13}C NMR spectrum of compound **2**

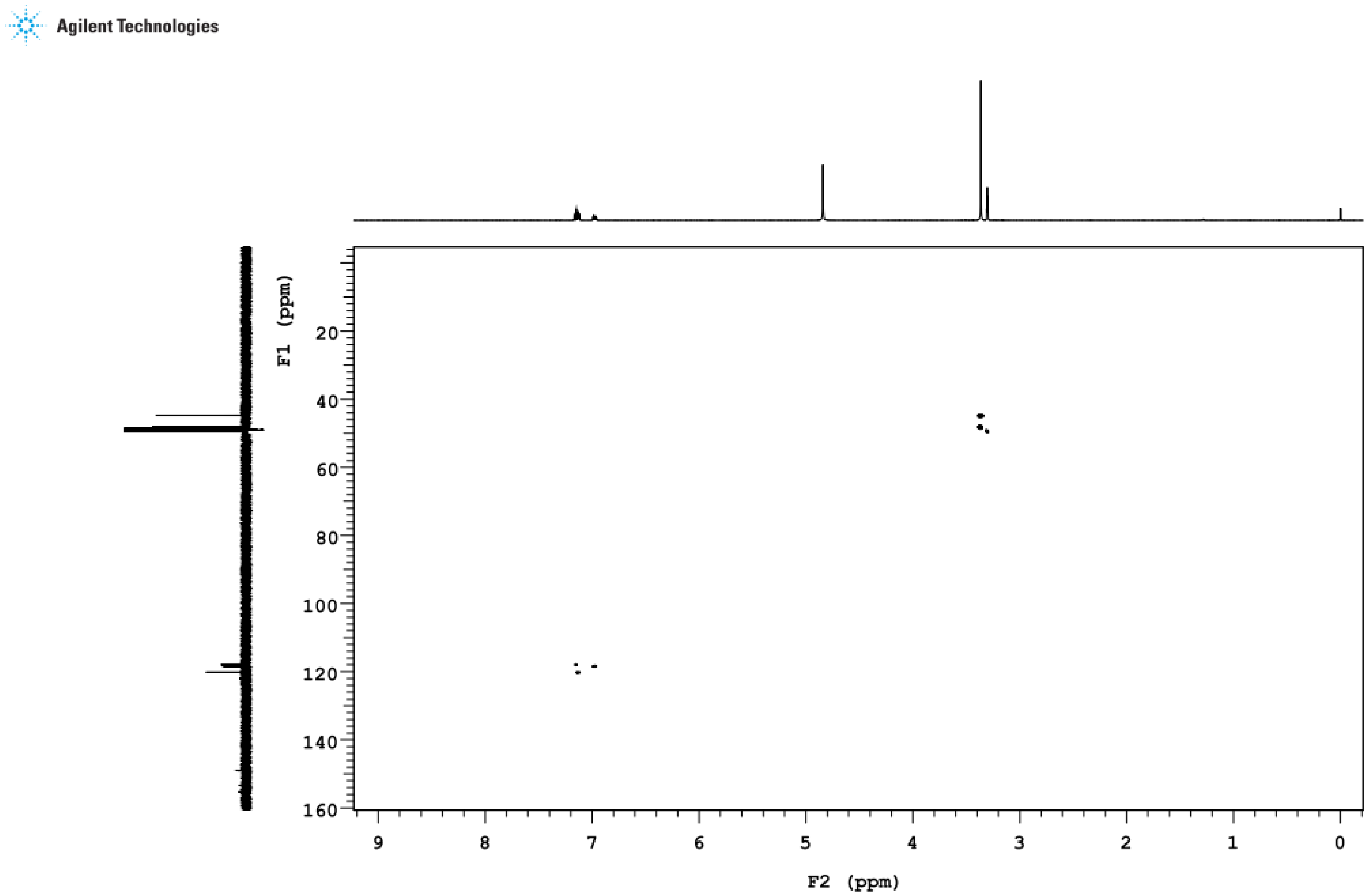

Fig. S9 The HSQC spectrum of compound **2**

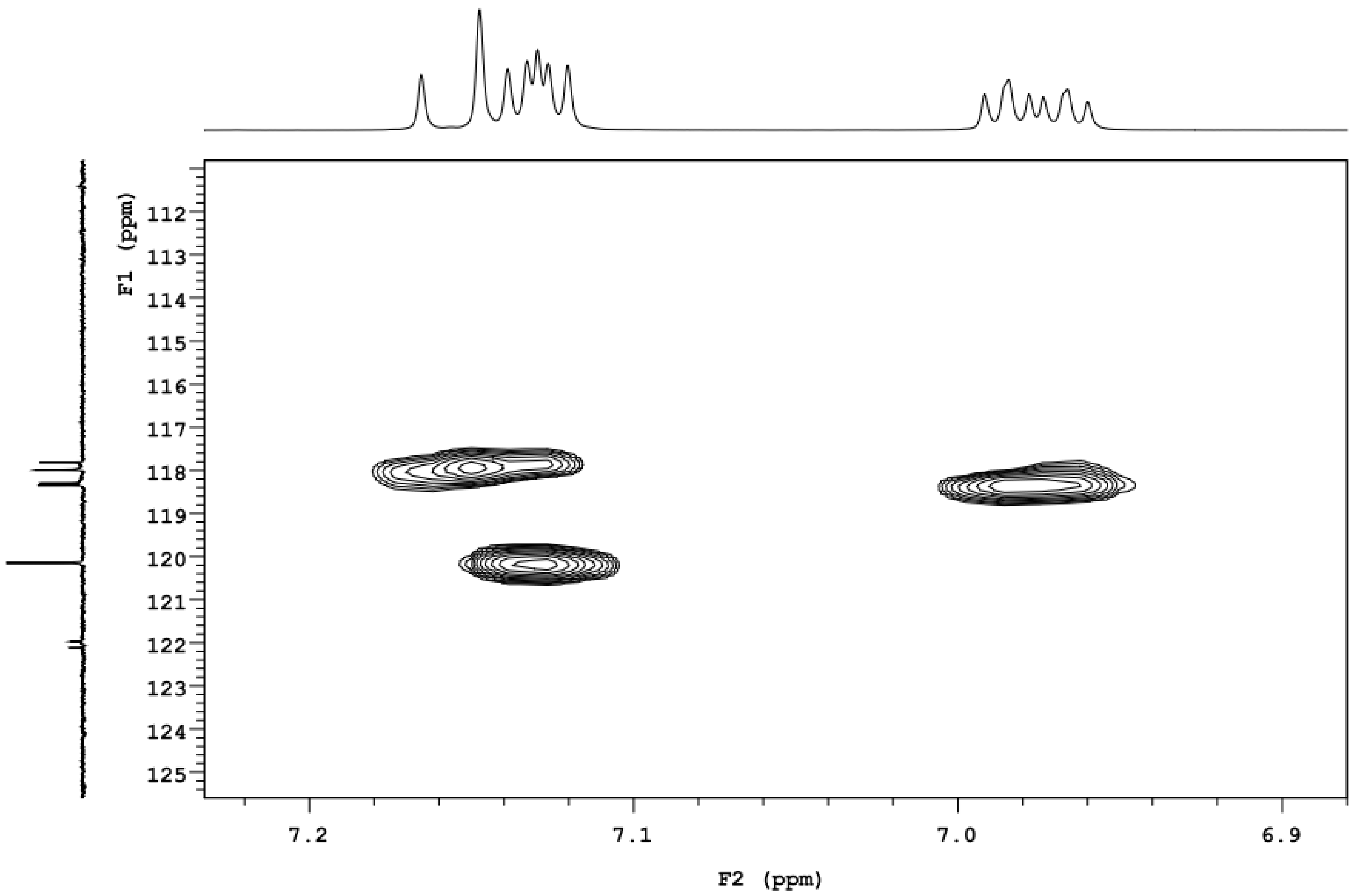

Fig. S10 The part of HSQC spectrum of compound **2**

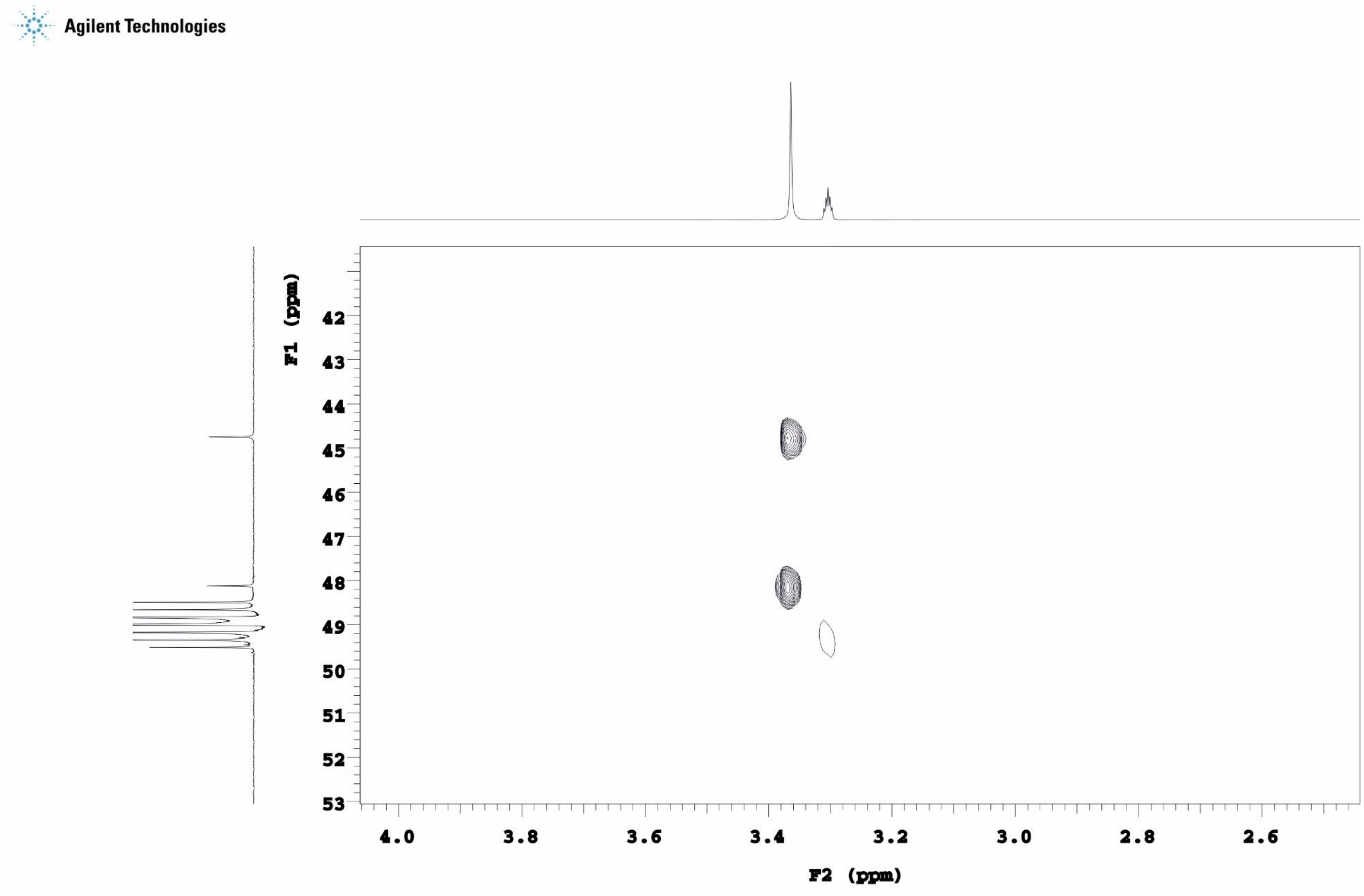

Fig. S11 The part of HSQC spectrum of compound **2**

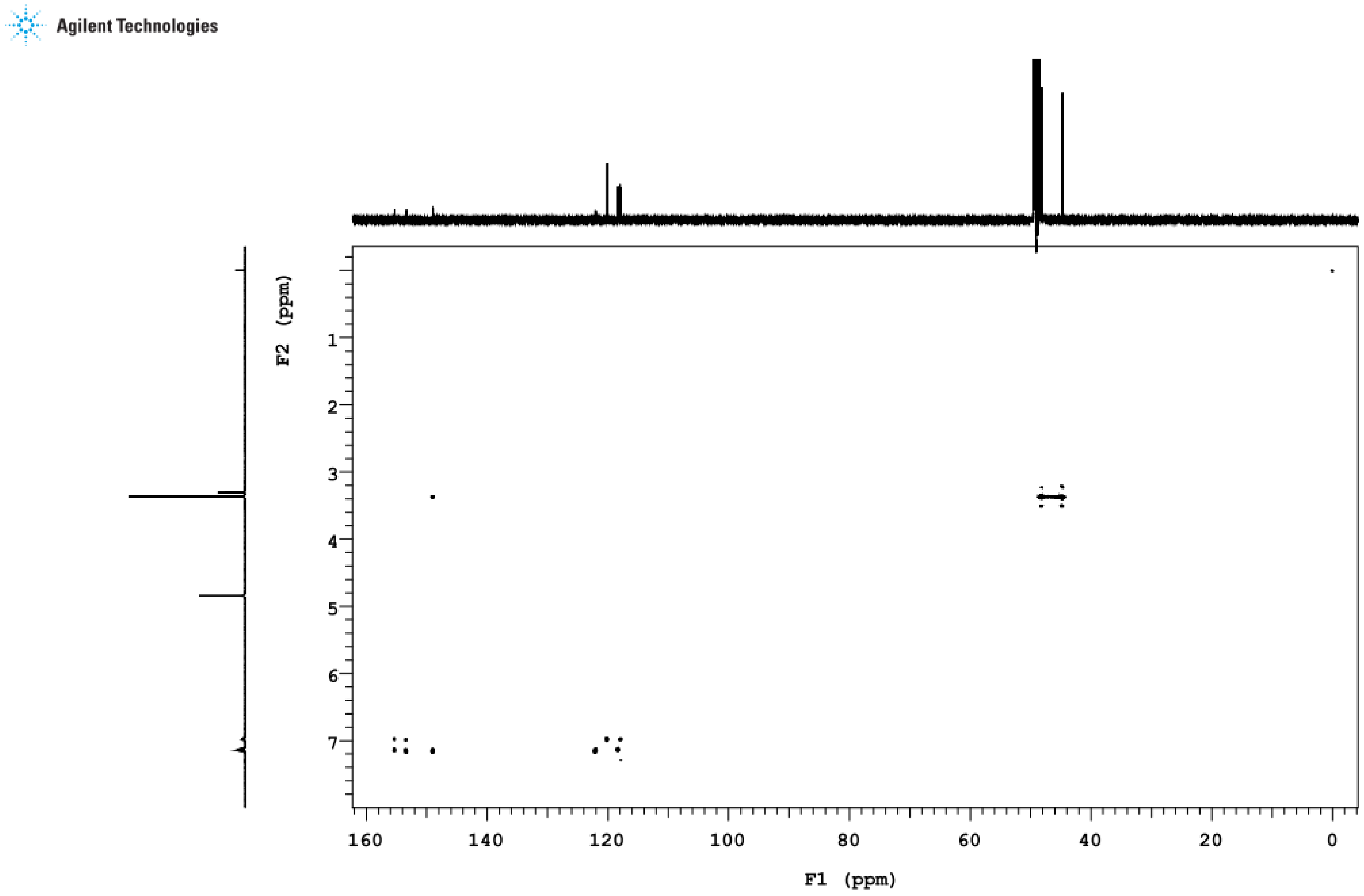

Fig. S12 The HMBC spectrum of compound **2**

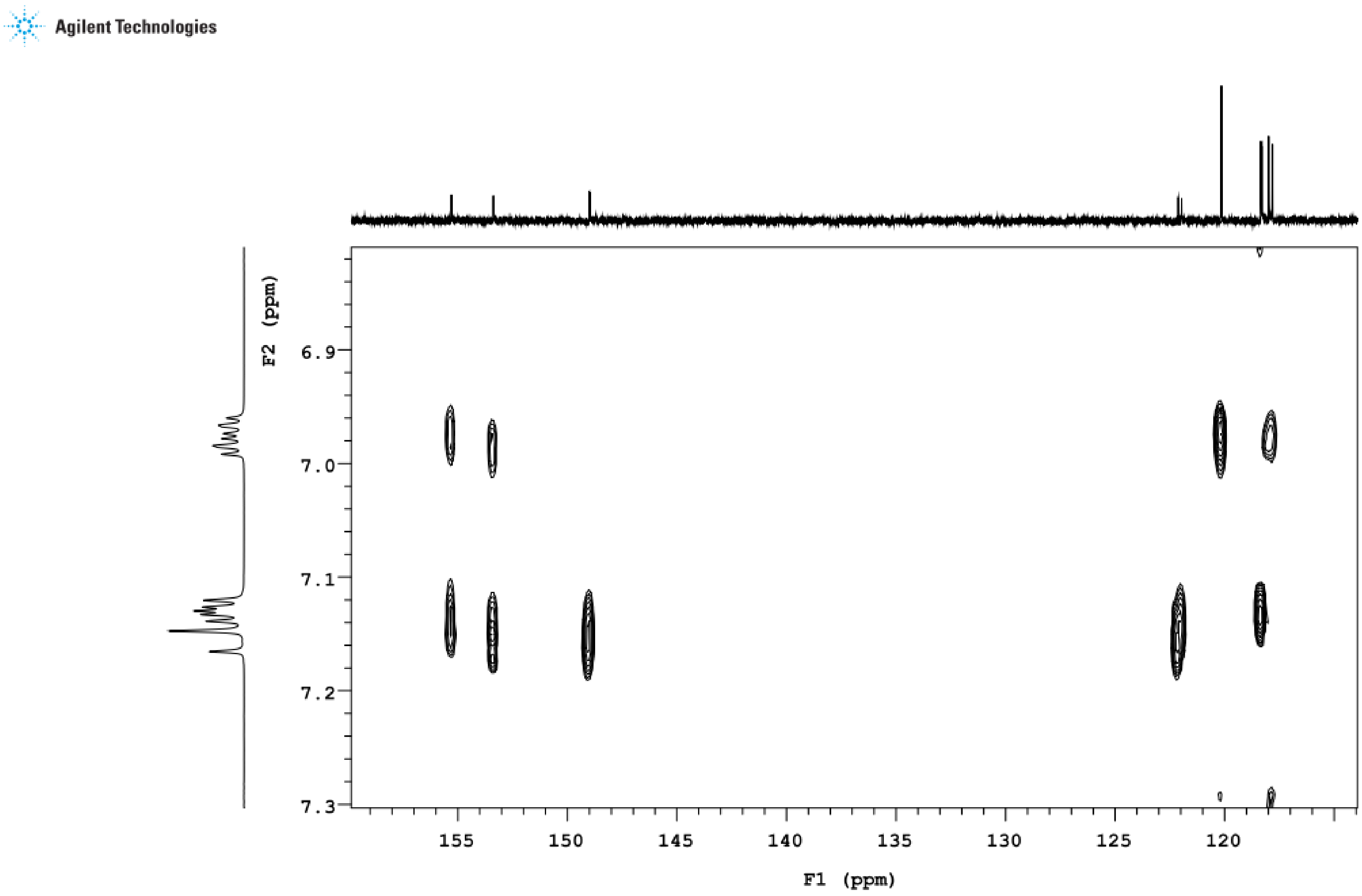

Fig. S13 The part of HMBC spectrum of compound **2**

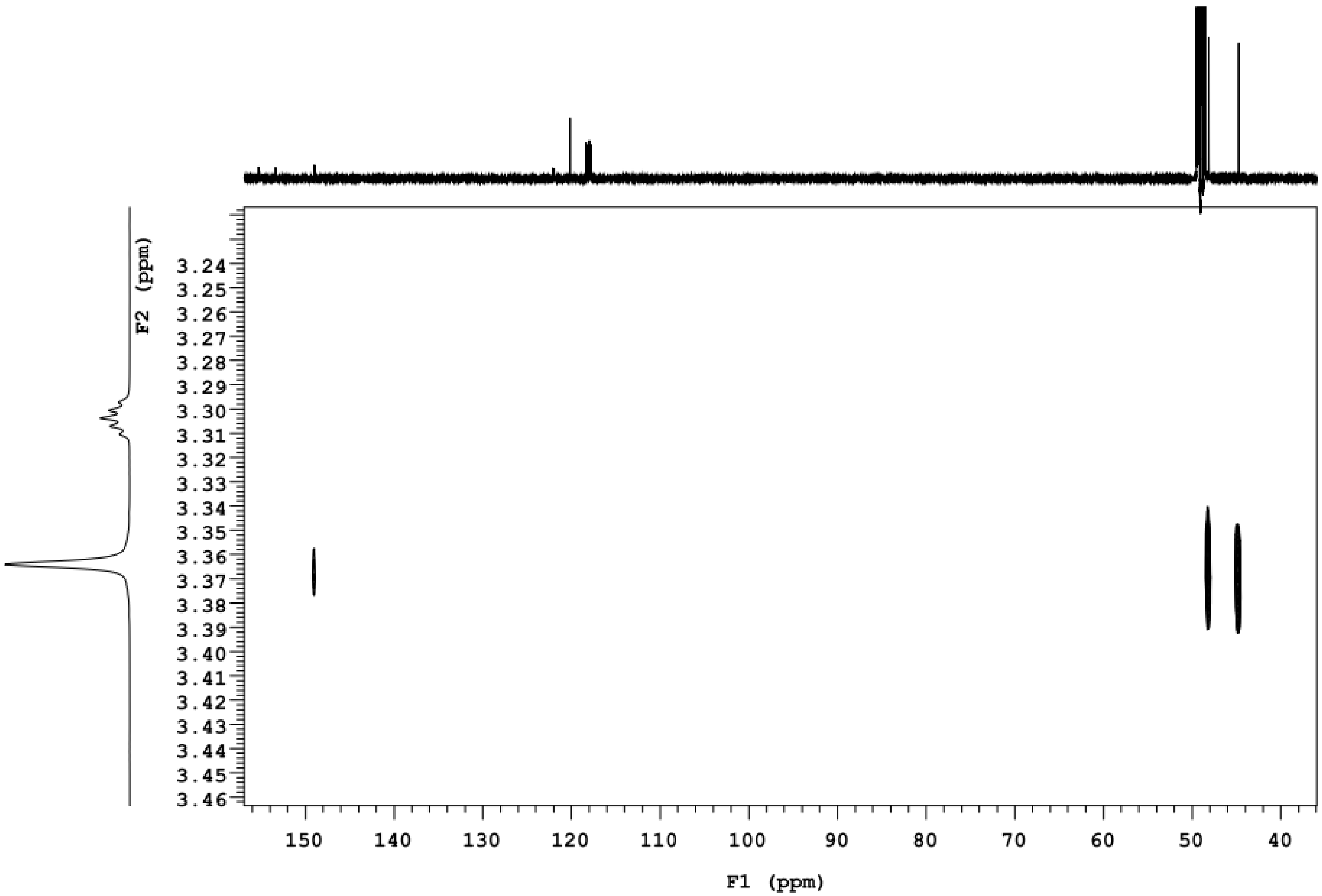

Fig. S14 The part of HMBC spectrum of compound **2**

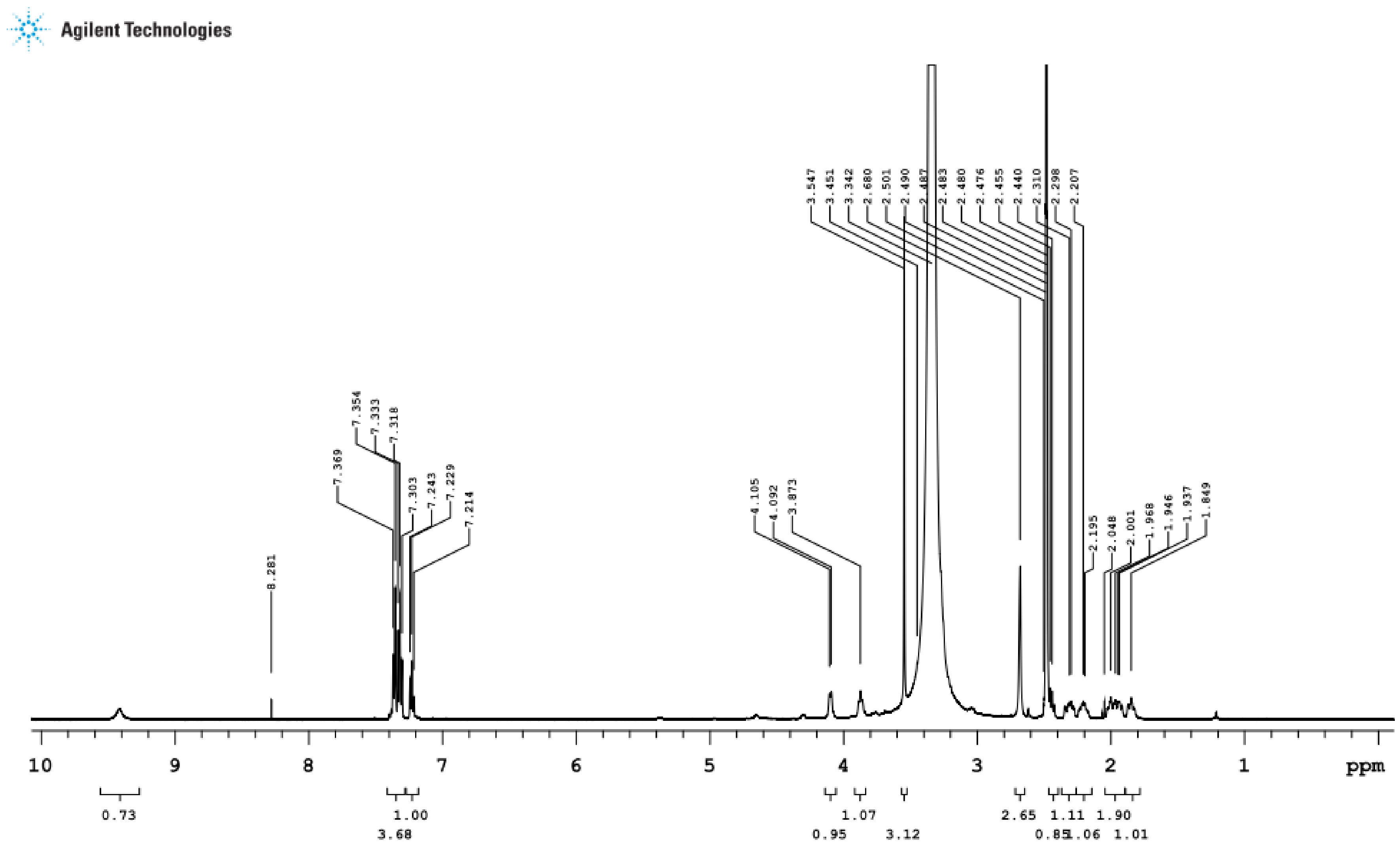

Fig. S15 The ^{1}H NMR spectrum of compound **3**

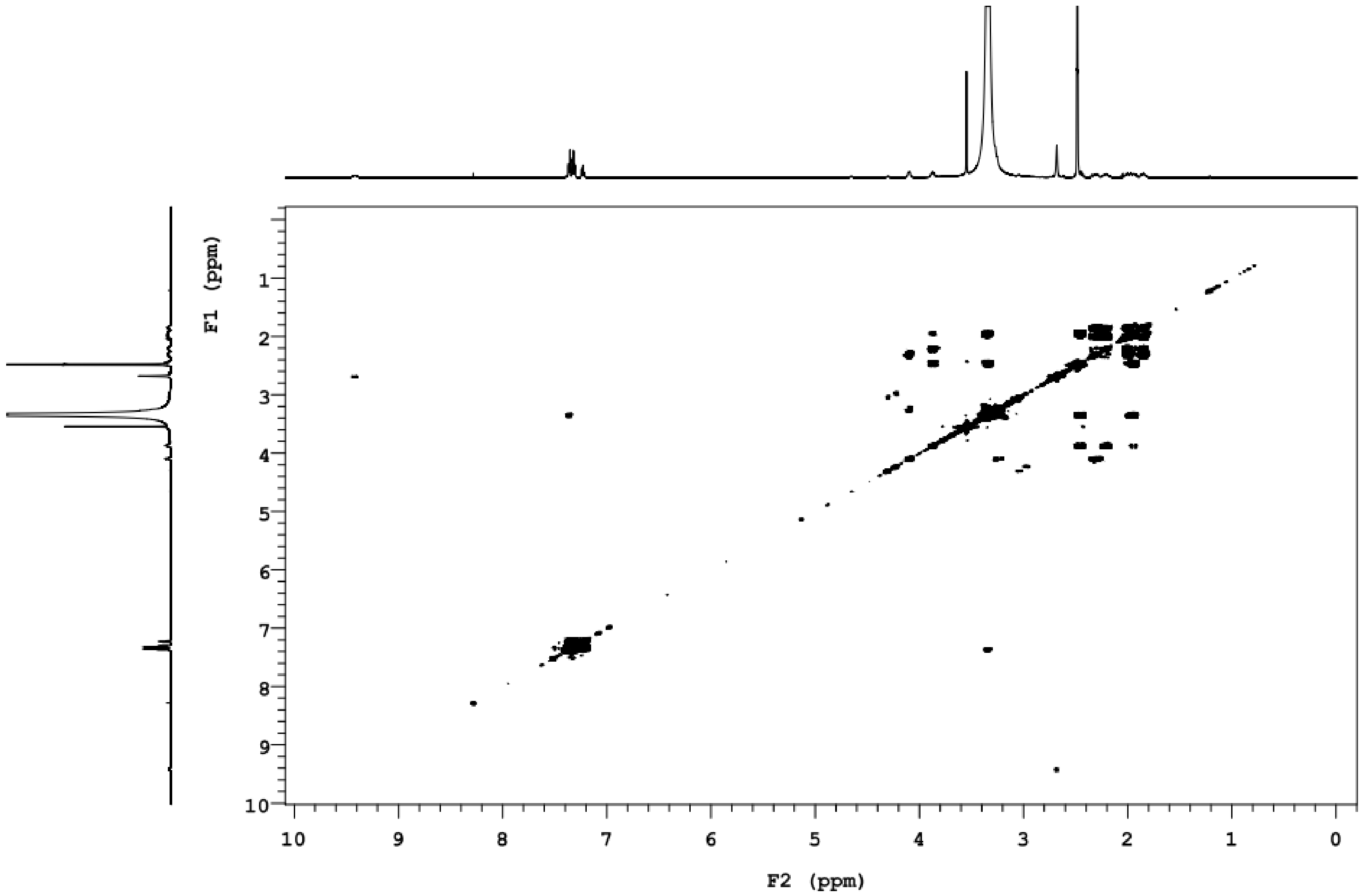

Fig. S16 The COSY spectrum of compound **3**

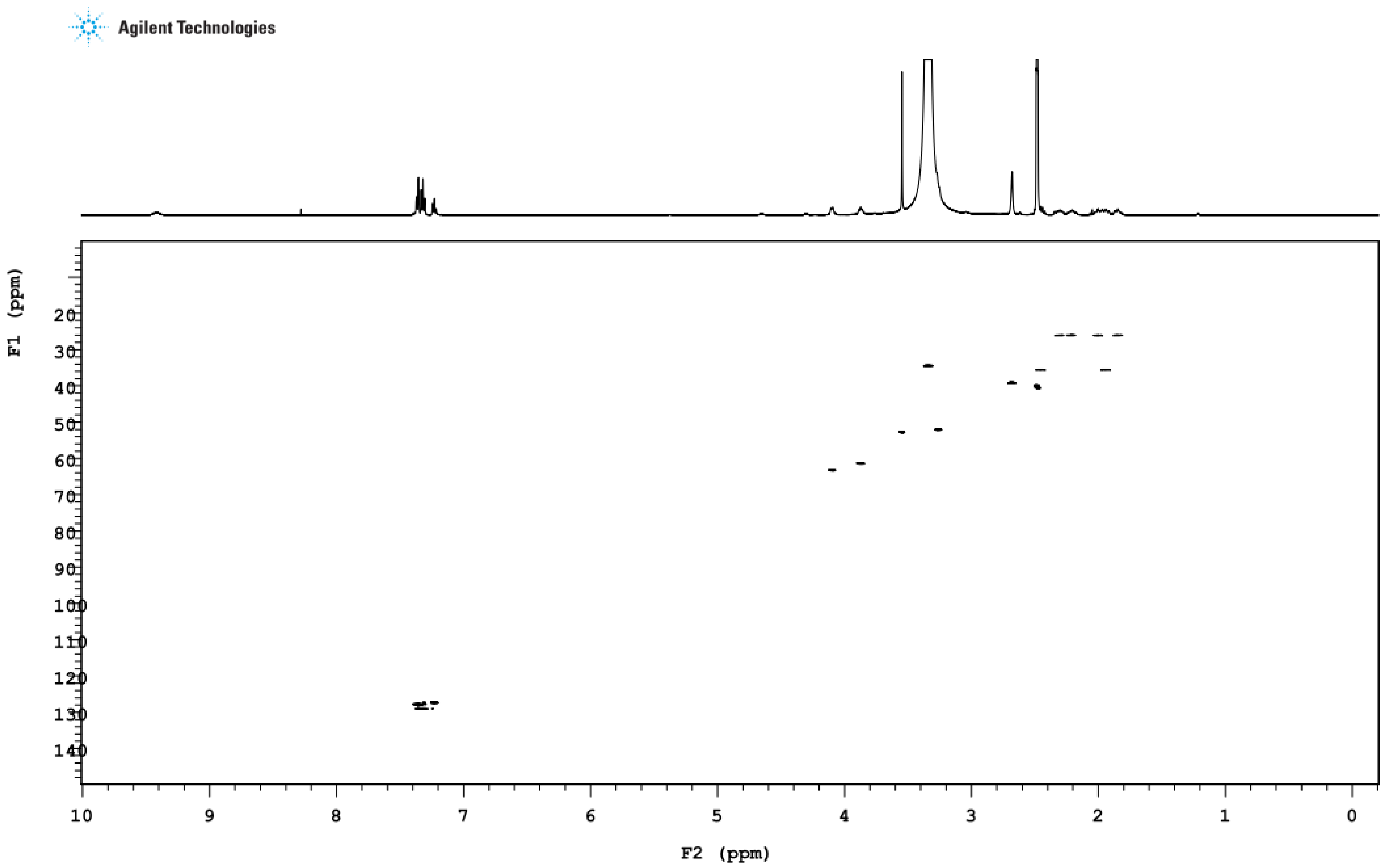

Fig. S17 The HSQC spectrum of compound **3**

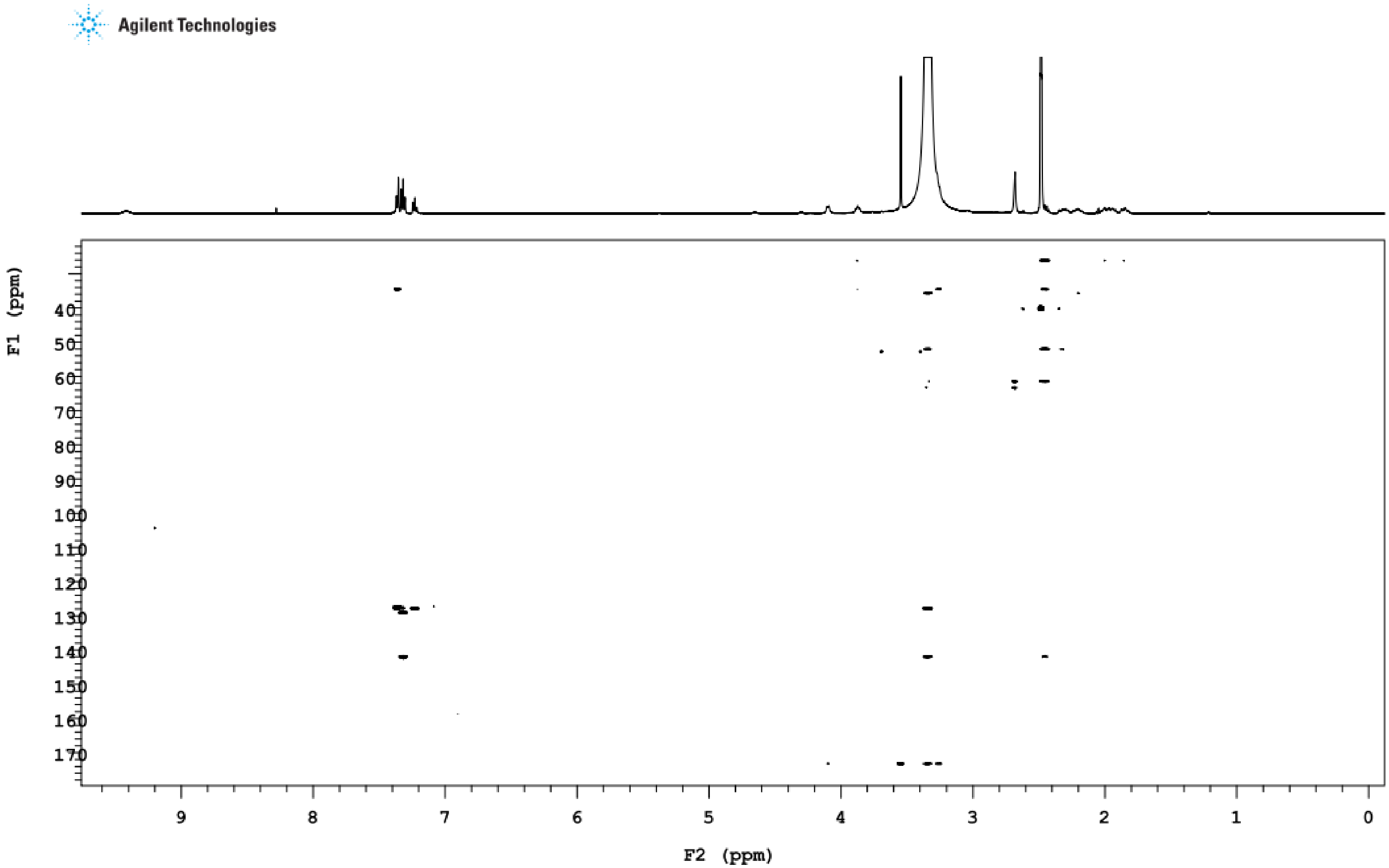

Fig. S18 The HMBC spectrum of compound
