# Supplementary material for: Identification and structural characterization of three psychoactive substances, phenylpiperazines (pBPP and 3,4-CFPP) and a cocaine analogue (troparil), in collected samples
Source: Forensic Toxicol. 2021 Sep 14;40(1):132–43. doi: 10.1007/s11419-021-00597-4 (PMC9715470; doi:10.1007/s11419-021-00597-4)
Supplement: Supplementary file 2 — Supplementary file2 (PDF 182 KB) [file 11419_2021_597_MOESM2_ESM.pdf]

Electronic Supplementary Material **ESM_2**

Forensic toxicology

Identification and structural characterization of three psychoactive substances: phenylpiperazines (*p*BPP and 3,4-CFPP) and cocaine analogue (troparil), in collected samples

Magdalena Popławska[1*], Elżbieta Bednarek[1*], Beata Naumczuk[1], Agata Błażewicz[1]

[1]National Medicines Institute, 30/34 Chełmska Street, 00-725 Warsaw, Poland,

*Corresponding author e-mail address:

m.poplawska@nil.gov.pl (Magdalena Popławska)

e.bednarek@nil.gov.pl (Elżbieta Bednarek)

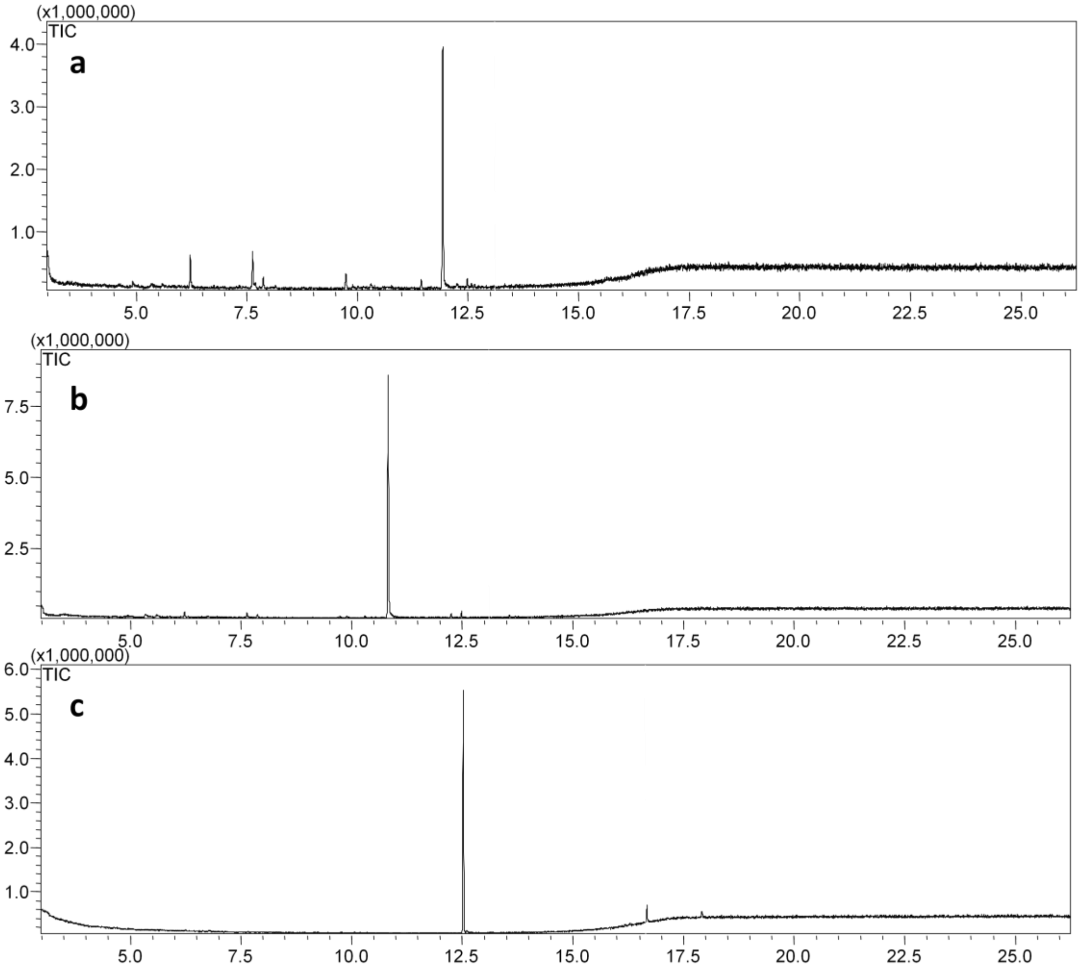

Fig. S19 Gas chromatography–mass spectrometry chromatograms of *p*BPP (compound **1**) (a), 3,4-CFPP (compound **2**) (b) and troparil (compound **3**) (c).

Collision energy optimization

When applying the collision energy (CE) gradient from 20 to 25 eV for values from *m/z* 200 to 400 the precursor ion at *m/*z 260.1653 was fragmented at a CE 21.5 eV. The predominant product ion in the MS/MS spectrum of compound **3** was observed at *m/z* 228.1391. However, relative intensities of all other product ions were below 3 %. When the final CE value at *m/z* 400 was elevated to 40 eV and according to the gradient troparil was fragmented at 26.0 eV, numerous product ions appeared on the MS/MS spectrum but their signals were still low. An increase of the initial CE setting to 35 eV (at *m/z* 200) and 40 eV at *m/z* 400 resulted in the extensive fragmentation of the main product ion via different CID pathways, giving many high signals on the MS/MS spectrum. When applying this last CE gradient program, product ion was fragmented at 36.5 eV. The higher CE favoured dehydrogenation process of the product ions. The signals at *m/z* ratio less by 2 Da compared to *m/z* of the fragments produced using lower CE were observed i.e. for the pair of product ions $C_5H_8N^+$ and $C_5H_{10}N^+$ at theoretical *m/z* 82.0655 and 84.0807 respectively, the ratio of their relative intensities were 0.4 at CE=21.5eV and 0.6 at CE=36.5eV, for the pair of product ions $C_{13}H_{14}N^+$ and $C_{13}H_{16}N^+$ at theoretical *m/z* 184.1119 and 186.1269 respectively, the ratio of their relative intensities were 1.2 at CE=21.5eV and 3.8 at CE=36.5eV. The similar trend could be observed for the following pairs of ions: at *m/z* 141.0692 and 143.0855; at *m/z* 115.0529 and 117.0689; at *m/z* 103.0545 and 105.0708.

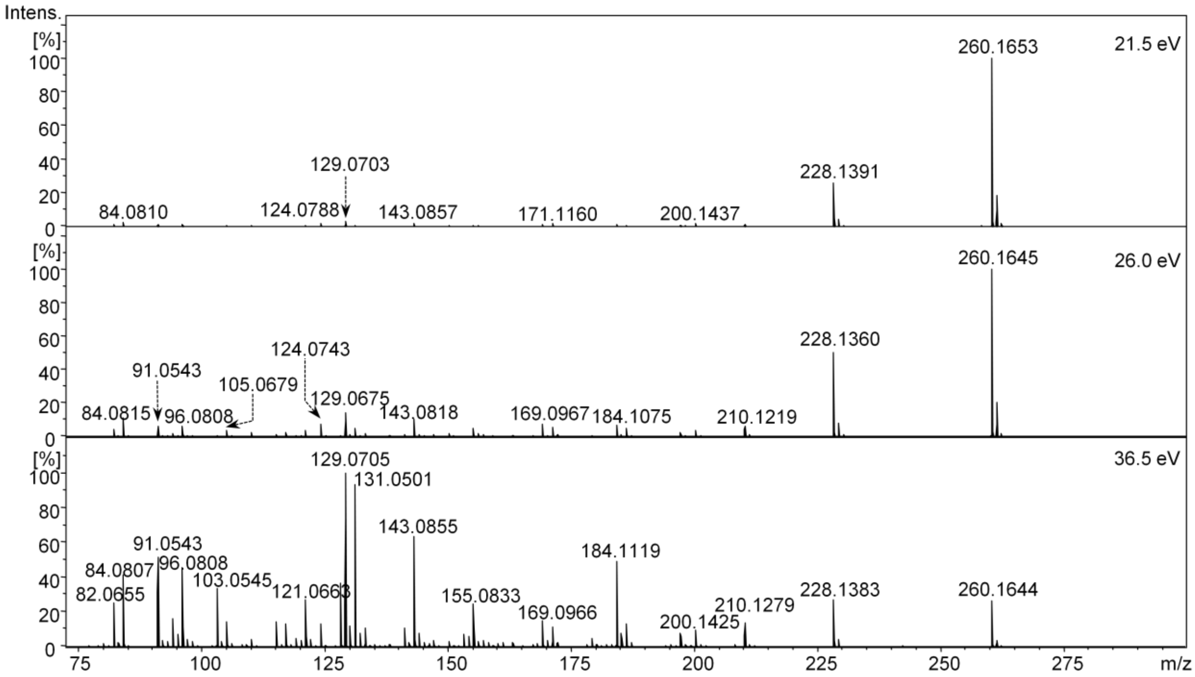

Fig. S20 Product ion mass spectra of troparil recorded at different collision energy gradient programs.
